# A Mixed Filtering Approach for Real-Time Seizure State Tracking Using Multi-Channel Electroencephalography Data

**DOI:** 10.1109/TNSRE.2021.3113888

**Published:** 2021-10-08

**Authors:** Alexander G. Steele, Sankalp Parekh, Hamid Fekri Azgomi, Mohammad Badri Ahmadi, Alexander Craik, Sandipan Pati, Joseph T. Francis, Jose L. Contreras-Vidal, Rose T. Faghih

**Affiliations:** Department of Electrical and Computer Engineering, University of Houston, Houston, TX 77004 USA, and also with the NSF IUCRC BRAIN Center, University of Houston, Houston, TX 77004 USA; Department of Electrical and Computer Engineering, University of Houston, Houston, TX 77004 USA; Department of Electrical and Computer Engineering, University of Houston, Houston, TX 77004 USA; Department of Biomedical Engineering, University of Houston, Houston, TX 77004 USA; Department of Electrical and Computer Engineering, University of Houston, Houston, TX 77004 USA; Department of Neurology, McGovern Medical School, The University of Texas Health Science Center at Houston, Houston, TX 77030 USA; Department of Biomedical Engineering and the Department of Electrical and Computer Engineering, University of Houston, Houston, TX 77004 USA; Department of Electrical and Computer Engineering, University of Houston, Houston, TX 77004 USA, and also with the NSF IUCRC BRAIN Center, University of Houston, Houston, TX 77004 USA; Department of Electrical and Computer Engineering, University of Houston, Houston, TX 77004 USA, and also with the NSF IUCRC BRAIN Center, University of Houston, Houston, TX 77004 USA

**Keywords:** Electroencephalography (EEG), epilepsy, Kalman filter, neurofeedback, real-time detection, state estimation, state-space methods

## Abstract

Real-time continuous tracking of seizure state is necessary to develop feedback neuromodulation therapy that can prevent or terminate a seizure early. Due to its high temporal resolution, high scalp coverage, and non-invasive applicability, electroencephalography (EEG) is a good candidate for seizure tracking. In this research, we make multiple seizure state estimations using a mixed-filter and multiple channels found over the entire sensor space; then by applying a Kalman filter, we produce a single seizure state estimation made up of these individual estimations. Using a modified wrapper feature selection, we determine two optimal features of mixed data type, one continuous and one binary analyzing all available channels. These features are used in a state-space framework to model the continuous hidden seizure state. Expectation maximization is performed offline on the training and validation data sets to estimate unknown parameters. The seizure state estimation process is performed for multiple channels, and the seizure state estimation is derived using a square-root Kalman filter. A second expectation maximization step is utilized to estimate the unknown square-root Kalman filter parameters. This method is tested in a real-time applicable way for seizure state estimation. Applying this approach, we obtain a single seizure state estimation with quantitative information about the likelihood of a seizure occurring, which we call seizure probability. Our results on the experimental data (CHB-MIT EEG database) validate the proposed estimation method and we achieve an average accuracy, sensitivity, and specificity of 92.7%, 92.8%, and 93.4%, respectively. The potential applications of this seizure estimation model are for closed-loop neuromodulation and long-term quantitative analysis of seizure treatment efficacy.

## I. Introduction

APPROXIMATELY 50 million people live with epilepsy worldwide [2]. In the US alone, the National Institutes of Health spends over $150 million each year on epilepsy research. This accounts for roughly 82% of research that is not coming from industry sources [3]. Epilepsy is a neurological disorder which can occur at any age, currently has no cure, and is characterized by seizures that can happen without noticeable warning [3]. This can lead to other health problems such as brain injury from falling, psychiatric conditions, and a reduction in quality of life [4], [5]. It is a spectrum with a wide range of seizure types, which vary from person-to-person [6], [7]. For example, a focal seizure is one that is triggered by a localized portion of the brain, while a general seizure can be triggered in multiple parts of the brain [7].

Treatments for epilepsy currently focus on anti-epileptic medications [8]. While most patients find that their symptoms are well controlled with a drug regimen, more than 90% still experience seizures [8], [9]. These medications also have various side effects such as: weight change, headaches, dizziness, and shaking [10], [11]. Independence is also a common concern among people living with epilepsy, with many questioning their ability to find employment, travel, or pursue higher education, even with treatment [11].

Due in part to the limited success of medications and because more invasive techniques such as surgical resection for focal seizures carry high risk, researchers are now exploring minimally invasive neurostimulation techniques such as Deep Brain Stimulation (DBS) and Vagus Nerve Stimulation (VNS) [12]–[15]. While long-term studies are still in progress, the results are promising, and both DBS and VNS have already achieved a higher rate of seizure reduction than medication-based interventions [14]–[16]. However, most of these devices are open-loop and work by applying constant or periodic stimulation without any feedback [17]. Open-loop systems have several limitations, such as lower battery life, reduced effectiveness of the treatment, and side-effects from stimulation such as disruption of cognitive function, dizziness, or mood changes [18]–[20]. The few closed-loop systems currently available are still limited and costly [21]. An ideal closed-loop system has several advantages including lower power consumption, reduction in stimuli, and fewer adverse effects to the treatment [18], [22]. Some of the challenges when closing the loop are measuring and decoding neural activity from the brain to detect the seizure.

While several methods of recording cortical activity exist, one of the most accessible and low-cost is electroencephalography (EEG). EEG is a non-invasive measurement of electrical activity produced by the brain and measured at the scalp [23]. Because of its safety, high temporal resolution, portability, high scalp coverage, recording reliability, and low power requirements, it is one of the preferred methods for recording brain activity [24], [25]. By selecting a non-invasive option for neural recording, the system becomes more widely accessible to the epileptic community, can be implemented quickly, and avoids the complications posed by invasive neural recording alternatives.

An effective closed-loop system must have accurate seizure detection or prediction because late intervention can be ineffective to prevent or terminate seizures early [26], [27]. Over the past decade, a number of solutions to seizure detection have been tested. The proposed solutions range from training neural networks, support vector machines, empirical mode decomposition, or wavelet transformation to determine the amplitude and frequency of the recorded signals [28]–[31]. While these methods use a wide range of approaches to predict a seizure, computationally intensive algorithms are required. This is not feasible in a system with limited resources, where minimizing power consumption and computational complexity is a high priority. Additionally, most of these approaches only result in the binary estimation, which provides no information about the progression of the seizure state as it evolves in time and space. Real-time estimation and monitoring of seizure state as they evolve and progress is clinically relevant as they may assist in developing feedback therapy (adaptive neuromodulation) [28], [29]. In this study, we define the likelihood of a seizure occurring as seizure probability. One practical application of seizure probability estimation for seizure abatement is with VNS, which can use real-time frequency and amplitude adaptation to better tailor treatment [32]. There are also clinical reasons why knowing seizure probability would be useful, such as determining efficacy of the neurostimulation treatment over the course of treatment or quantitatively determining the impact of medication on seizures [33], [34].

Seizures are emergent neural phenomena characterized by multiple state transitions starting from the interictal to preictal, ictal onset, propagation, termination, and the postictal state [35]. When estimating the seizure state, model selection is incredibly important. In this case, a state-space model is preferred as it can be related to the underlying biology and the output is continuous, rather than binary, which provides information as to the seizure evolution and severity [1], [36]. Furthermore, state-space modeling algorithms are computationally efficient and can model complex systems to a high degree of accuracy, making them ideal for low resource systems [37]. Expanding on previous work [1], where the authors employed a mixed-filter approach to perform seizure state estimation using two EEG channels, we propose that the removal of the self-imposed channel count restriction may lead to increases in accuracy and robustness of the state-estimation and be more applicable to non-focal type seizures [7]. Then, we employ a mixed-filter approach to incorporate both binary and continuous features. While we can estimate the seizure state using a single estimation alone, a combined continuous estimation uses multiple channels, so it is less susceptible to sensor noise, provides us with higher accuracy, and includes information on the severity of a seizure [36], [38], [39]. By having information about the seizure probability, the amplitude and the frequency in DBS and VNS applications can be automated in a real-time closed-loop manner. Thus, accurately estimating seizure states may improve treatment and reduce the possibility of serious brain injuries [40], [41].

## II. Methods

**Fig. 1.**
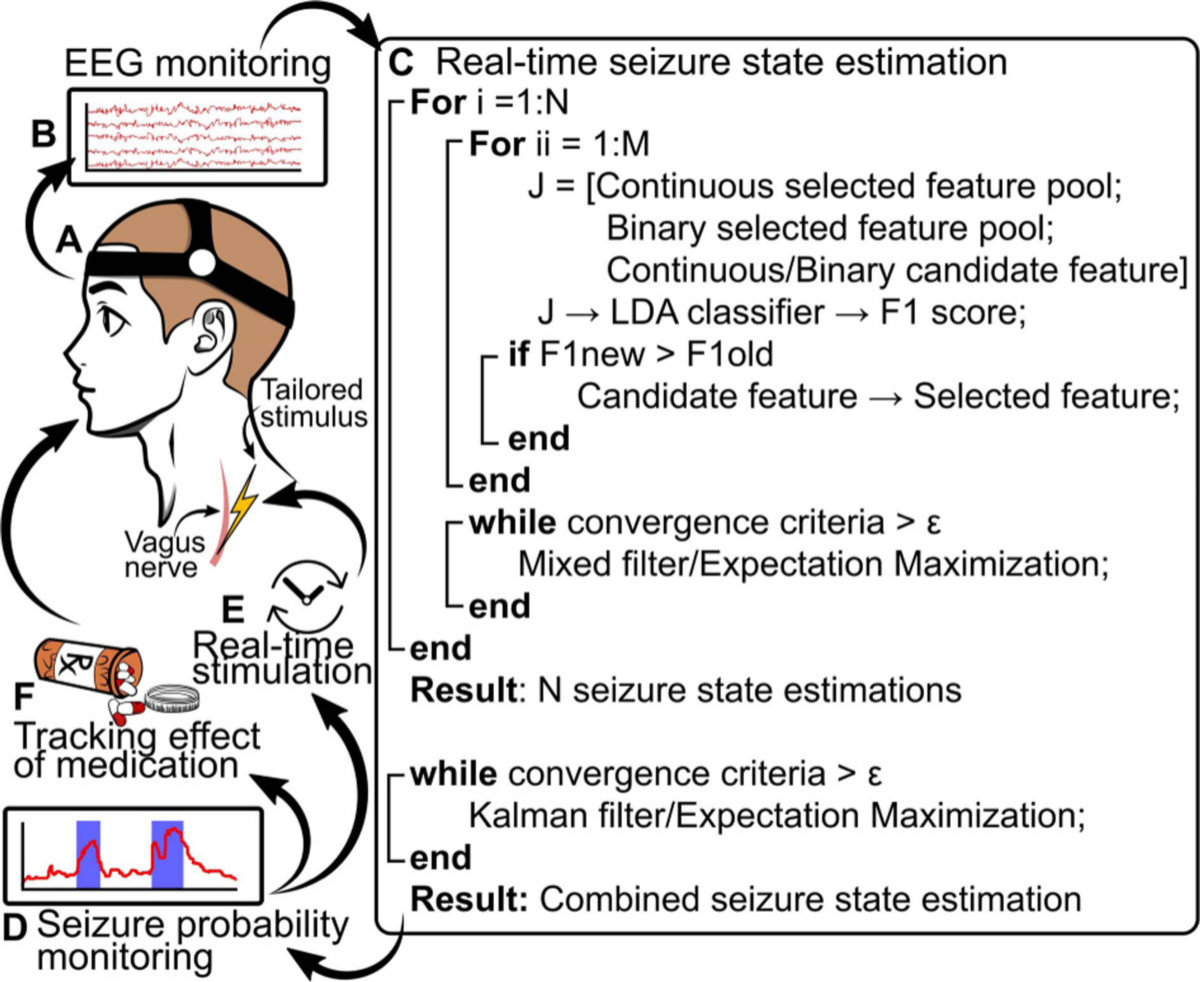
Procedure for real-time seizure estimation. A person wearing a EEG headset **A**, which monitors neural activity **B**. This information is then passed to the seizure state estimation algorithm **C** which returns the seizure state severity estimation **D**. This work has several future applications. Some examples are the real-time tailoring and activation of neural stimulation modalities such as VNS/DBS **E** and monitoring efficacy of anti-seizure medications or other treatments **F**.

Figure 1 outlines the proposed method and some future uses for finding a combined seizure state estimation, which we define as a seizure state estimation made up of N number of individual estimations using 2*N* features. A person wearing an EEG headset has their neural activity recorded in real-time. Using a Linear Discriminant Analysis (LDA) classifier we find two features, one continuous and one binary, that best estimate the seizure state. A mixed-filter with expectation maximization (EM) is applied to make a single estimation of the seizure state. EM is an iterative process that finds maximum-likelihood estimates for model parameters when the data is incomplete or has unobserved latent variables [36], [42]. This process is repeated *N* times where *N* = 5 is the maximum number of iterations and 2*N* is the number of features that make up the estimation. A square-root Kalman filter is employed to include information from multiple predictions. A Kalman filter is a recursive filter that estimates the hidden state of a linear dynamic system from a series of noisy measurements [43]. The square-root Kalman filter returns a combined seizure state estimation which is made up of the *N* individual estimations.

Having multiple estimations is useful because, if one of the selected features is contaminated with noise, this could lead to poor performance. By using a filter with multiple inputs, if one of the features is noisy, the filter can still provide a relatively accurate estimation using the other features [44]–[47]. The output of this process is a single seizure state estimation with high accuracy. Parts (E) and (F) in Figure 1 highlight two examples of future applications of this work, such as real-time tailoring of closed-loop neural stimulation (E) and tracking the effectiveness of anti-seizure medications (F).

### A. Data

The data set (CHB-MIT Scalp EEG Database) was collected in 2010 at the Boston Children’s Hospital [48], [49]. It consists of continuous high density EEG recordings from 22 pediatric subjects (5 males, ages 3–22, and 17 females, ages 1.5 – 19) with intractable seizures for several days following the cessation of anti-seizure medication to determine if surgical intervention was warranted.

The EEG recordings have between 46 to 52 electrodes, which were placed according to the international 10–20 system of EEG electrode positions. All signals were recorded at 256 Hz sampling frequency. Recordings were accomplished using a bipolar montage, where each channel consists of two electrodes. The database consists of 916 hours of EEG recordings and the data set contains 182 seizures over the entire subject population. Further information on the original data collection can be found in [48], [49].

### B. Feature Extraction

For feature extraction, the EEG data of a single subject was divided into three data sets: a training set, a validation set, and a testing set. The first session with a recorded seizure within the subjects data set was used as the training set, the session with the second recorded seizure was used for the validation set, and the remaining seizure containing sessions are used as the testing data set [1]. By splitting the training, validation, and testing data in these proportions, the number of seizures needed to train the model is reduced, which limits the necessary training data collection efforts and sets a lower limit to the effectiveness of this model. Additionally, segmenting the data this way provides a larger testing set, so we can better determine the performance of our approach.

Due to the session-to-session variability, the EEG features were normalized with a min/max normalization based on the statistical characteristics of the feature derived from the first minute of each session. The first minute of data was also selected for this normalization process to maintain the real-time applicability of this method. An assumption that is made when using this mixed filter is that the continuous feature must be monotonic in relation to the unknown seizure state [50]. However, EEG signal information is oscillatory in nature, so it is not possible for the amplitudes to be monotonic in relation to the seizure state [51], [52]. One way to resolve this issue is using EEG band powers (i.e. monotonic) as the candidate features.

Using the Fast Fourier Transform (FFT) with a sliding one second window, each channel is decomposed into the absolute power of four characteristic EEG band powers: Delta (0.1–4 Hz), Theta (4–7 Hz), Alpha (8–15 Hz), and Beta (16–31 Hz). Gamma band (31+ Hz) was excluded from the candidate bands because Gamma band is more likely to be contaminated with noise from muscles. Moreover, previous work has found that lower frequency bands provide more information about the seizure state [53].

#### 1) Continuous Feature:

For the continuous feature, the raw feature values are converted from amplitude squared to dB so as to enforce having a positive amplitude square estimation from the model.

#### 2) Binary Feature:

The binary feature extraction process is a slightly modified version of the continuous feature extraction protocol. The raw EEG feature data is binarized using an LDA classifier, which eliminates the need to apply the dB transformation.

### C. Feature Selection

To determine the best continuous and binary features, a modified wrapper feature selection is utilized that consists of an (i) LDA, which is a simple linear computationally-efficient classifier that is used to create a predictive model, (ii) F1 score as an evaluation method, (iii) greedy forward selection for subset selection, and (iv) a stopping criteria of using 2*n* features where *n* = 1, 2, 3*, . . . , N* = 5 is the total number of estimations [54].

Using two pools of candidate features, one binary pool and continuous pool, at each iteration, the modified wrapper feature selection algorithm selects one candidate feature from the continuous pool, is used to train an LDA classifier with any previously selected features, then repeats this process for the binary pool. The selected continuous and binary features are then placed into their respective candidate pools.

Feature selection was done by training *M* LDA classifiers with *J* features, where *M* is the number of features in the candidate pool and *J* is the number of features in the selected feature pool plus one feature from the candidate feature pool. Each LDA classifier is tested using the validation set. The resulting arrays are then compared to the true binary seizure state [55], [56]. The validation set is used to find features that are generalizable and have the power to estimate the hidden seizure state. Without using a validation set, there is a possibility that our model would be overfit and would not be able to make an accurate estimation. Changing the stopping criteria from previous paper [1], which was set to one, helps to reduce the inter-feature correlation since candidate LDAs now use the previously selected features as well as one candidate feature to determine if the candidate feature improves the performance.

For the binarization of each selected binary feature, an LDA is trained using each of that selected binary features on both the training and validation sets. The misclassification cost was again derived based on the proportion of the duration of seizure to non-seizure data in the training and validation sets. The same LDA is then used to binarize the selected binary feature on the test set. Performing the binarization after finding the best features was done to reduce the computational costs as well as increasing the accuracy of the binarization because we can use both the training and validation sets for the training of the binarization LDAs. If binarization occurred prior to feature selection, using both the training and validation set would bias the LDAs used to test the performance to the binary features.

To compare the level of accuracy each feature has, F1 scores are compared rather than absolute accuracy. Using accuracy alone, a feature could have high accuracy because it is biased toward specificity. Instead, the *F*1 score is employed due to its emphasis on the effect of precision and recall, which is given in the following equation,

(1) $$F1=2\times \frac{\left(precision\times recall\right)}{precision+recall}$$

**Fig. 2.**
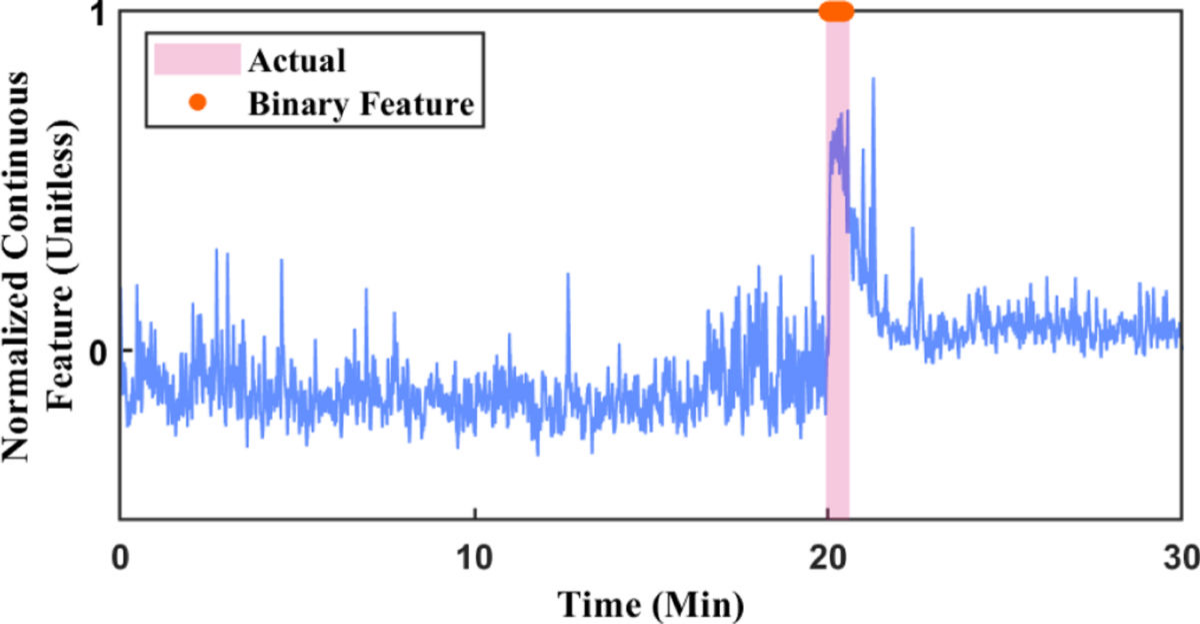
First Pair of Features Selected for Subject 1, Session 4. The shaded area marks the period designated as the true seizure state. While the binary feature alone (orange dots) has high accuracy, the continuous feature (blue curve) provides additional information about the seizure probability.

An example of this process presented in Figure 2, which shows the pair of features that best predict the seizure state. The binary feature (orange dots) detects only the true seizure state (purple shaded region), while the continuous feature (blue curve) has higher activity around the seizure state. This indicates that both the binary and continuous features are effective in estimating the seizure state and its severity.

### D. Seizure State Modeling

The model of the seizure state relies on the assumption that a continuous seizure state *x*_*k*,*n*_ at *k* time step, can be represented as the following first-order auto-regressive state-space process [38], [52], [57]–[60],

(2) $$x_{k,n}=\rho_{n}x_{k-1,n}+\eta_{k,n}$$

where *n* = 1, 2, . . . , *N* = 5 is the number of estimations, 0 < *ρ_n_* < 1 is the forgetting rate parameter for estimation *n*, and *η*_*k*,*n*_ ∼ *N*(0, $\sigma_{\eta ,n}^{2}$) is the independent Gaussian random variable that represents the process noise for estimation *n*, where $\sigma_{\eta ,n}^{2}$ is its variance.

### E. Seizure State Estimation

The observation model for the continuous feature is the power of a frequency band, *r*_*k*,*n*_ (amplitude squared). As the filter requires *r*_*k*,*n*_ to be unbounded, the continuous feature is converted from amplitude squared to dB by taking 10*log*(*r*_*k*,*n*_) to form *v*_*k*,*n*_ such that

(3) $$v_{k,n}=\log\left(r_{k,n}\right)=\alpha_{n}+\beta_{n}x_{k,n}+\epsilon_{k,n}$$

where $\alpha_{n}$ governs the baseline value of the continuous feature when the subject is not experiencing any seizure state at estimation *n*, $\beta_{n}$ is the level of the continuous feature as a function of the seizure state, and the continuous measurement noise parameter $\epsilon_{k,n}$ is an independent Gaussian random variable, i.e. $\epsilon_{k,n}∼N\left(0,\sigma_{\epsilon ,n}^{2}\right)$, with the unknown variance $\sigma_{\epsilon ,n}^{2}$.

Besides the continuous feature, the binary feature is assumed to follow a Bernoulli distribution such that,

(4) $$Pr\left(\lambda_{k,n}|x_{k,n}\right)={\left(p_{k,n}\right)}^{\lambda_{k,n}}{\left(1-p_{k,n}\right)}^{1-\lambda_{k,n}}$$

where $\lambda_{k,n}$ is derived from the raw amplitude squared data at estimation *n* and is selected using the process described in section II-C. The observation model is defined as:

(5) $$p_{k,n}=\frac{e^{\mu_{n}+x_{k,n}}}{1+e^{\mu_{n}+x_{k,n}}}$$

The logistic function is chosen for this specific filter design because the probability function, *p*_*k*,*n*_, must be bounded between zero and one. Additionally, the seizure probability *p*_*k*,*n*_ can be inferred by its relationship with the seizure state *x*_*k*,*n*_. The parameter *μ_n_* is estimated individually for each subject from chance probability, *p_chance_*, where *p_chance_* is the probability of a seizure occurring [38], [61]. The chance probability used in the model is selected as the duration of seizure activity in both the training and validation set, divided by the total duration of the combined sets [38], [61].

The mixed filter treats the seizure status as a hidden biophysical state and results in a continuous seizure state estimation. In addition to making an estimation about the occurrence of a seizure, continuous estimation also provides information about the severity of the seizure. This filter was selected because it incorporates both continuous and binary features, which better estimates the hidden state [54], [62].

### F. Expectation Maximization Algorithm

To estimate the hidden continuous seizure state, the EM algorithm is used on the combined data from the training and validation sets to estimate the unknown state-space parameters:

(6) $$\theta_{n}=\left(\rho ,\alpha ,\beta ,\sigma_{\eta }^{2},\sigma_{\epsilon }^{2},x_{k}\right)$$

where ***ρ***, ***α***, ***β***, $\sigma_{\eta }^{2}$, $\sigma_{\epsilon }^{2}$, and ***x****_k_* stand for the *N* × 1 column vectors including the values of *ρ_n_*, *α_n_*, *β_n_*, $\sigma_{\eta ,n}^{2}$, $\sigma_{\epsilon ,n}^{2}$, and *x*_*k*,*n*_ for all *n* estimations.

The EM process is an iterative method to find the maximum likelihood of (*θ_n_*) by alternating between the expectation and maximization steps [63], [64]. During the expectation step, the algorithm creates a log-likelihood function from the initial values of the parameters. Then the maximization step finds the values for these parameters that maximize the log-likelihood function [36], [65]. These resulting parameters are then used in the log-likelihood function and the process is repeated until convergence [66], [67]. Next, the parameters and features found with the training and validation sets were applied to the model to track the continuous seizure state in the test set. For this portion of testing, the forward filter was used exclusively as the backward smoother is not real-time applicable because it requires knowledge of future measurements to improve the estimation of the present measurements [68]–[70]. The formulation for the EM algorithm is presented in the supplementary information.

### G. Kalman Filter

As the mixed filter employs a single continuous feature and a single binary feature to return a single estimation, the resulting matrix is now composed of *n* rows. When *n* > 1, the Kalman filter takes the multiple estimations and produces a combined estimated seizure state. The combined seizure estimation model is defined with a linear state-space framework such that *z_k_* is a hidden combined seizure state at time *k* = 1, 2, . . ., *K*:

(7) $$z_{k}=Az_{k-1}+\omega_{k}$$

(8) $$x_{k}=B+Cz_{k}+v_{k}$$

where *A* is the unknown state transition scalar. **B** is the unknown bias vector of *N* length, where *N* is the total number of estimations and **B**(*n*) is the *n*−*th* value of vector **B**. **C** is the unknown measurement transition vector, which estimates the variance for each input. *ω_k_* ∼ *N*(0, ${\text{Σ}}_{\omega }^{2}$) and ν*_k_* ∼ *N*(0, ${\text{Σ}}_{v}^{2}$) are independent unknown noise variance matrices with Σ*_ω_* and Σ*_ν_* unknown noise covariances associated with the process and measurements noises, respectively. The formulation for the Kalman filter algorithm is presented in the supplementary information.

### H. Square-Root Kalman Filter

The formulation of the Kalman filter does not provide the numerical stability needed for this application [71], [72]. For this reason, an implementation of a square-root covariance filter algorithm was required [72] to ensure that the error covariance matrix of the state estimate of *z_k_*, **Σ***_k_*, will always yield a symmetric non-negative matrix that is well conditioned. The formulation for the square-root Kalman filter can be found in the supplementary information.

The unknown state-space parameters are calculated using the EM algorithm outlined in section II-F without the added binary feature modification:

(9) $$\theta_{\phi }=\left(A,B,C,{\text{Σ}}^{2}{}_{\omega },{\text{Σ}}^{2}{}_{\nu }\right)$$

Taking all *n* separate estimations as an input matrix, the square-root Kalman filter algorithm returns a single vector representing the combined state estimation as shown using pseudo-code in Fig. 1. After each time and measurement update pair, the process is repeated with the a posteriori estimates to update the model and predict the new a priori estimates.

### I. Binary Seizure Estimation

Using the continuous seizure state estimation from the training and validation sets, a final LDA was trained to binarize our data. Taken together, the selected features, the trained binarization LDA, and the state-space parameters are then applied to the test set. The output is a binarized form of the seizure state estimation. This provides performance statistics with which to compare the above algorithm with past attempts as all past attempts to classify this data did so with binary classes. The sensitivity, accuracy, and specificity of our approach are then compared to the performance of other approaches. The formulation for binary seizure estimation is presented in the supplementary information.

## III. Results

To reduce the inter-feature correlation, improve F1 score, or both, we modified the feature selection method from the previous work [1]. Table I presents the change in correlation coefficient and F1 score of the two best features, one continuous and one binary, using the previous feature selection method, where the best features were found individually for each subject and the proposed method. The proposed method instead attempts to find the best combination of features for each subject. By reducing correlation, the chance of multicollinearity is reduced, which can increase the variance of the coefficient estimates and makes the estimates very sensitive to minor changes in the model [73]–[75]. As expected, the correlation between the selected features using the proposed method decreased in all the subjects with the exception of subjects 1 and 7, which saw no change in feature correlation. The F1 score increased for all the subjects with the exception of subject 8, which saw no change.

**Fig. 3.**
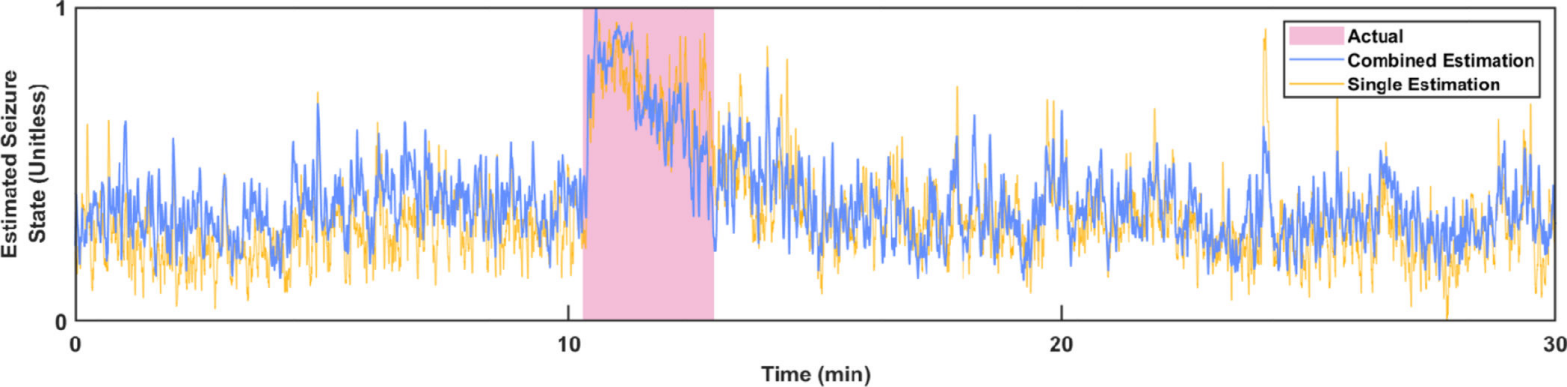
Estimated Seizure State for Subject 8, Session 4. Combined seizure state estimation and the first individual estimation from this process. The shaded area marks the period designated as the true seizure state.

An example of the continuous seizure state estimation using five estimations is depicted in Figure 3. The purple highlighted area marks the occurrence of the actual seizure state as labelled from the original data set. The blue line is the continuous sample-by-sample estimation from the combination of the five estimations, the first estimation is plotted in yellow, and creates a pronounced seizure state that matches well with the true seizure state. Next, the seizure state binarization LDA was trained using the test set from the EM algorithm to produce a binary seizure state estimation.

Table II presents the results of the proposed method compared to previous studies. The proposed method obtains an average accuracy of 92.7%, average sensitivity of 92.8%, and average specificity of 93.4%. This is an increase in average accuracy of 6.9%, an increase in average sensitivity of 1.3%,and an increase in average specificity of 7.7% when compared to the previous work [1].

Table III highlights the effects of increasing the number of estimations to create the combined seizure estimate has on the performance metrics for all ten subjects. On average, as we increase the number of estimations, we see an increase in performance. Additionally, after three estimations, the returned increase in performance starts to diminish.

**Fig. 4.**
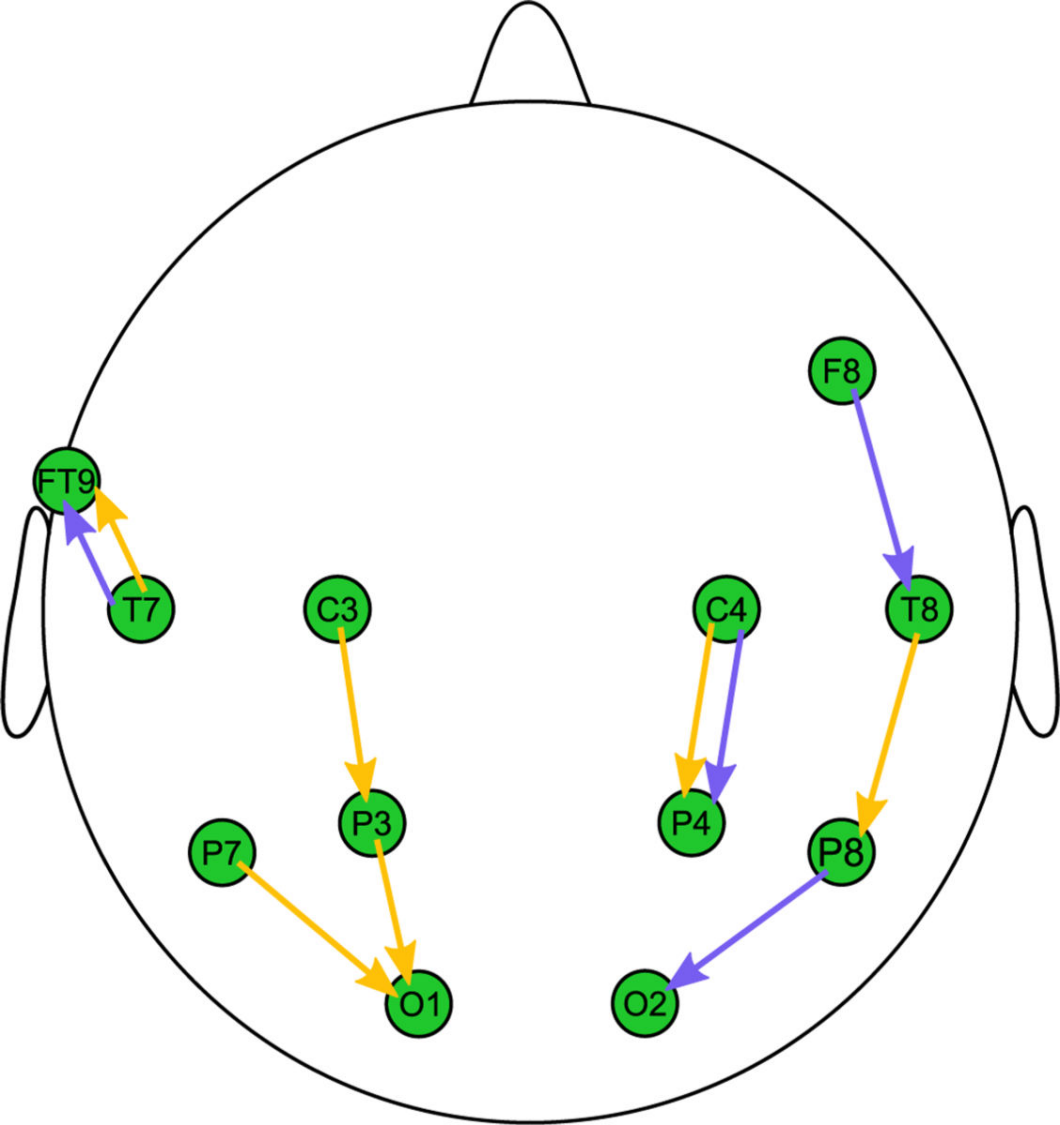
Subject 1 scalp map Sensor pairs selected for the continuous features are shown in blue, while binary features are shown in yellow.

Figure 4 shows the selected bipolar sensors for Subject 1 where the start of the arrow represents the positive sensor and the arrowhead represents the negative sensor. The selected continuous features are represented using blue arrows and the binary features are represented with yellow arrows. In some instances, multiple bands for a feature type, continuous or binary, were selected using the same electrode pair. In these cases, only a single arrow was used. Scalp maps for subjects 2 through 10 can be found in the supplementary information along with a table of sensor pairs used and frequency bands selected for all features and subjects.

## IV. Discussion

By combining seizure state estimations, the accuracy, sensitivity, and specificity of the total estimation are increased as compared to a single estimation. In [48] and [76], the authors used feature vectors as inputs for an SVM classifier, whereas, [77] used an LDA for estimation. The feature vectors used by all three are also more computationally demanding than the proposed method. For example, [48] uses temporal and spectral statistics, [76] uses fuzzy entropy, and [77] calculates wavelet coefficients. All three of these studies classify in segments of time instead of estimating on a point-by-point basis which limits response speed, the resolution, and the real-time applicability of their methods. Lastly, all three of these studies perform binary estimation exclusively. This means that any information regarding state transition during seizure evolution and seizure probability is lost.

The method proposed here demonstrates the feasibility of real-time continuous estimation for the detection of seizure events with a minimum amount of training data. Further studies utilizing multiple electrode pairs should be investigated to evaluate the suitability of our method for patients with for multifocal seizures. As it derives a continuous estimation, the method also provides information on the seizure probability, which can potentially be used as a quantitative way to measure efficacy of the chosen intervention. Furthermore, by understanding the severity of a seizure, treatment can be adapted for real-time seizure mitigation applications, such as VNS and DBS, where the frequency and amplitude can be adjusted depending on the severity of the seizure. While the results of the proposed method achieve comparable performance to other studies [48], [76], [77], this method uses a more computationally efficient method that can be applied in a real-time manner and provides information on seizure probability.

When compared to the previous work [1], we find an improvement for accuracy, sensitivity and specificity across subjects with the exception of subject 10. One probable explanation for this is measurement noise either in the training or validation set. The presence of noise in either of data sets not only can cause feature selection to select non-consistent and noisy features, but also is problematic for finding unknown parameters of mixed filters and Kalman filter. One way to avoid this is using more data for training and validation, however, this requires more recordings from each subject. Alternatively, by using adaptive filtering such as H-infinity and dedicated sensors to measure ocular and EMG artifacts, the measurement noise could be reduced while still maintaining real-time applicability [78], [79]. As shown in Table III, using only a single estimation resulted to best performance for subject 10. Features for the training, validation, and the first seizure of the test set of subject 10 can be found in the supplementary information.

Because this research was performed using an existing data set, future work should aim at verifying the methods using real-time detection of a seizure state in a clinical setting. Further testing could be done using alternative artifact removal methods to improve the quality of the EEG recordings, which should improve the performance of this method. Additional work should determine the relationship of the likelihood of a seizure occurring, which we have defined here as seizure probability, with the clinical measures of seizure intensity to determine if there is a correlation. However, since this measure tracks seizure probability it is more likely to track with the occurrence of clinically reported seizures.

Further research should also focus on determining the optimal number of estimations for a subject and seizure type. This could be done by determining the accuracy of each estimation prior to the application of the mixed-filter. It would be beneficial to determine the relationship between individual estimation accuracy and its impact on the combined estimation of the seizure state. This would provide a threshold to the accuracy of an estimation and quantitatively determine the optimum number of estimations for the combined estimation.

The mixed filter uses a single continuous and a single binary estimation. Due to this limitation, it was necessary to apply a Kalman filter algorithm to determine the combined estimation. A more computationally efficient method may modify this base filter to accept more than a single continuous and binary input. This would eliminate the need for the Kalman filter and could increase the speed of estimations, which is one of the main limiting factors in real-time seizure estimation [26], [27]. The proposed method indicates that it is possible to perform real-time seizure detection using EEG and that by removing the channel limitation from the previous work [1] we can make multiple estimations from pairs of features across several channels that can improve the overall performance of the estimation. This continuous estimation can be used in a closed-loop system with frequency or amplitude modulation to reduce the severity of the seizure.

## V. Conclusion

In this study, we presented a method for using multiple features that best describe a seizure. From the entire sensor space, we determined one continuous and one binary feature to produce a single seizure state estimation. We repeated this process to produce multiple estimations and by applying a Kalman filter, we obtained a combined seizure state estimation which is made up of the multiple estimations. The resulting combined seizure state estimation has higher accuracy, sensitivity, and specificity than we could obtain using a single estimation.

## Supplementary Material

supp1-3113888

## Figures and Tables

**TABLE I:** Correlation and F1 Score of the Best Pair of New Features Using Our New Feature Selection Method and the Best Pair of Features Selected Using the Previous Method [1]

	Correlation		FI Score	
Subject	*	[1]	*	[1]
1	64.0	64.0	82.5	63.6
2	41.3	53.8	9.0	6.5
3	37.9	61.1	82.7	54.6
4	71.0	75.0	21.8	17.0
5	77.5	88.5	96.4	88.5
6	49.0	57.0	34.4	21.8
7	54.0	54.0	91.8	84.5
8	3.0	64.0	56.8	56.8
9	64.6	70.0	98.3	96.4
10	49.0	57.0	95.3	64.3

Average	51.1	64.4	66.9	55.4
S.D.	20.1	10.3	10.2	29.6

* indicates results obtained through the current study

**TABLE II:** A Comparison Between Studies That Analyzed the Same Data Set. Here We Compare Accuracy, Specificity, and Sensitivity Values of Our Proposed Method to Other Studies

Performance Statistics											

	Accuracy				Sensitivity				Specificity		
Subject	*	[1]	[77]	[78]	*	[1]	[49]	[77]	*	[1]	[77]
1	98.3	94	99	94	96.1	92.7	100	99	100	94.1	99
2	98.1	75.1	100	80	92.2	100	100	100	98.1	75.1	100
3	86.6	82	98	95	94.5	91.4	100	97	86.4	81.8	98
4	99.0	89.9	97	77	99.6	93.3	100	96	99.7	89.9	98
5	98.4	96.8	98	76	98.8	98.2	74	98	98.5	96.8	98
6	63.1	61.1	96	74	79.9	78	86	96	63.5	61.1	96
7	99.4	86.6	97	84	88.2	84.9	100	97	100	86.6	97
8	96.5	75.8	96	81	99.9	95.3	100	96	99.9	74.7	96
9	99.8	96.6	96	88	99.1	95.9	100	95	99.9	96.6	96
10	87.9	99.6	98	73	79.9	85.7	100	98	88.0	99.8	96

Average	92.7	85.8	97.5	82.2	92.8	91.5	96	97.2	93.4	85.7	97.4
S.D.	10.9	11.6	1.3	7.5	7.35	6.4	2.7	1.5	11.1	11.8	1.4

* indicates results obtained through the current study.

**TABLE III:** The Change in Performance Measures for All Subjects as a Function of the Number of Estimates Used

Number of Estimates															

	One			Two			Three			Four			Five		
Subject	Acc	Sen	Spe	Acc	Sen	Spe	Acc	Sen	Spe	Acc	Sen	Spe	Acc	Sen	Spe
1	94.4	94.1	96.0	96.4	93.2	98.1	98.1	94.3	98.9	98.3	96.1	100	98.3	96.1	100
2	75.1	75.1	100	94.6	80.4	94.7	94.4	82.2	94.5	97.8	91.1	97.8	98.1	92.2	98.1
3	82.9	91.2	82.9	83.1	91.2	83.0	84.1	91.8	84.1	85.6	94.9	85.4	86.6	94.5	86.4
4	89.9	92.3	88.9	89.7	94.0	89.6	90.3	93.8	90.3	99.0	97.6	99.7	99.0	99.6	99.7
5	96.9	96.8	96.7	97.5	97.6	97.6	98.1	98.4	98.2	98.2	98.6	98.4	98.4	98.8	98.5
6	61.4	78.4	61.6	61.4	79.1	62.8	62.2	78.7	62.2	62.7	79.1	62.7	63.1	79.9	63.5
7	86.6	84.9	86.8	98.4	84.1	100	98.4	84.1	100	99.2	87.4	100	99.4	88.2	100
8	88.7	93.7	88.4	96.1	99.8	99.8	96.1	99.5	99.5	96.3	99.4	99.4	96.5	99.9	99.9
9	96.9	95.9	96.9	99.6	96.1	99.6	99.8	96.3	99.8	99.8	98.4	99.9	99.8	99.1	99.9
10	99.5	76.4	99.8	80.5	78.1	80.8	84.8	82.6	84.8	83.0	84.2	83.0	87.9	79.9	88.0

Average	87.2	87.9	89.8	89.7	89.4	90.6	91.6	90.2	91.2	92.0	92.7	92.6	92.7	92.8	93.4
S.D.	11.1	8.0	10.9	11.3	7.8	11.4	10.9	7.2	11.3	11.3	6.6	11.7	10.9	7.4	11.1
