# Supplementary material for: A Mixed Filtering Approach for Real-Time Seizure State Tracking Using Multi-Channel Electroencephalography Data
Source: IEEE Trans Neural Syst Rehabil Eng. Author manuscript; Available in PMC 2021 Nov 26. (PMC8626138; doi:10.1109/TNSRE.2021.3113888)
Supplement: supp1-3113888 [file NIHMS1747033-supplement-supp1-3113888.pdf]

A Mixed Filtering Approach for Real-Time Seizure State Tracking Using Multi-Channel Electroencephalography Data

Alexander G. Steele, Sankalp Parekh, Hamid Fekri Azgomi, Mohammad Badri Ahmadi, Alexander Craik
Sandipan Pati, Joseph T. Francis, Jose L. Contreras-Vidal, and Rose T. Faghih

I. Expectation Maximization

To build the continuous seizure state estimation model, the EM algorithm is used on the combined data from the training and validation sets to estimate the unknown state-space parameters:

$$\boldsymbol{\theta}_n = (\boldsymbol{\rho}, \boldsymbol{\alpha}, \boldsymbol{\beta}, \boldsymbol{\sigma}_\eta^2, \boldsymbol{\sigma}_\epsilon^2, \boldsymbol{x}_k) \tag{1}$$

where $\boldsymbol{\rho}$, $\boldsymbol{\alpha}$, $\boldsymbol{\beta}$, $\boldsymbol{\sigma}_\eta^2$, $\boldsymbol{\sigma}_\epsilon^2$, and $\boldsymbol{x}_k$ stand for the $N \times 1$ column vectors including the values of ρ_n, α_n, β_n, $\sigma_{\eta,n}^2$, $\sigma_{\epsilon,n}^2$, and $x_{k,n}$ where $n = 1, 2, ..., N$ is the number of estimations, $0 < \rho_n < 1$ is the forgetting rate parameter for estimation n, and $\eta_{k,n} \sim N(0, \sigma_{\eta,n}^2)$ is the independent Gaussian random variable that represents the process noise for estimation n, where $\sigma_{\eta,n}^2$ is the variance of the process noise for estimation n. α_n governs the baseline value of the continuous feature when the subject is not experiencing any seizure state for estimation n, β_n is the level of the continuous feature as a function of the seizure state at estimation n, and the continuous measurement noise parameter $\epsilon_{k,n}$ is an independent Gaussian random variables, i.e. $\epsilon_{k,n} \sim N(0, \sigma_{\epsilon,n}^2)$, with the unknown variance $\sigma_{\epsilon,n}^2$.

The EM process is an iterative method to find the maximum likelihood of $\boldsymbol{\theta}_n$ by alternating between the expectation and maximization steps [2], [3]. During the expectation step, the algorithm creates a log-likelihood function from the initial values of the parameters. Then the maximization step finds the values for these parameters that maximize the log-likelihood function [4], [5]. These resulting parameters are then used in the log-likelihood function and the process is repeated until convergence [6], [7]. Next, the parameters and features found with the training and validation sets were applied to the model to track the continuous seizure state in the test set. For this portion of testing, the forward filter was used exclusively as the backward smoother is not applicable in real-time because it requires knowledge of future measurements to improve the estimation of the present measurements [8]–[10]. The EM algorithm is as follows:

1) *Expectation Step*: At $(l+1)^{th}$ iteration of the algorithm, the expectation of data log likelihood in the estimation step is computed given the selected continuous and binary features and $\boldsymbol{\theta}_n^{(l)}$, which contains the parameter estimates from the nth estimation and the $(l)^{th}$ iteration.

   a) *Forward Filter*: The seizure probability variable, $x_{k,n|k,n}$ given $\boldsymbol{\theta}_n^{(l)}$, is estimated using a recursive non-linear filter algorithm [7], [11], [12]. Here the notation $k, n|m, n$ denotes the expectation of the state variable at k given responses up to observation m for estimation n.

$$x_{k,n|k-1,n} = \rho_n^{(l)} x_{k-1,n|k-1,n} \tag{2}$$

$$\sigma_{k,n|k-1,n}^2 = \rho_n^{2(l)} \sigma_{k-1,n|k-1,n}^2 + \sigma_{\eta,n}^{2(l)} \tag{3}$$

$$C_{k,n} = (\beta_n^{2(l)} \sigma_{k,n|k-1,n}^2 + \sigma_{\epsilon,n}^{2(l)})^{-1} \sigma_{k,n|k-1,n}^2 \tag{4}$$

$$x_{k,n|k,n} = x_{k,n|k-1,n} + C_{k,n}[\beta_n^{(l)}(v_{k,n} - \alpha_n^{(l)} - \beta_n^{(l)} x_{k,n|k-1,n} + \sigma_{\epsilon,n}^{2(l)})\lambda_{k,n} - p_{k,n|k,n}] \tag{5}$$

$$\sigma_{k,n|k,n}^2 = [(\sigma_{k,n|k-1,n}^2)^{-1} + p_{k,n|k,n}(1 - p_{k,n|k,n}) + (\sigma_{\epsilon,n}^{2(l)})^{-1} \beta_n^{2(l)}]^{-1} \tag{6}$$

   b) *Backward Smoother*: Equations 5 and 6 result in the posterior mode estimates $x_{k,n|k,n}$ and the variance for the estimation $\sigma_{k,n|k,n}^2$, respectively. A fixed interval smoothing algorithm is employed to compute $x_{k,n|K,n}$ and $\sigma_{k,n|K,n}^2$ [13]. The algorithm is as follows [11]:

$$x_{k,n|K,n} = x_{k,n|k,n} + J_k(x_{k+1,n|K,n} - x_{k+1,n|k,n}) \tag{7}$$

This paper was presented in part at the proceedings of the Asilomar Conference on Signals, Systems, and Computers [1]. Correspondence should be addressed to senior author Rose T. Faghih.

Alexander G. Steele, Sankalp Parekh, Hamid Fekri Azgomi, Alexander Craik, Jose L. Contreras-Vidal, and Rose T. Faghih are with the Department of Electrical and Computer Engineering at the University of Houston, Houston, TX 77004 USA (e-mail: agsteele, sparekh2, hfekriazgomi, arcraik, jlcontreras-vidal, rtfaghih@uh.edu). Alexander G. Steele, Jose L. Contreras-Vidal, and Rose T. Faghih are also with the NSF IUCRC BRAIN Center at the University of Houston. Mohammad Badri Ahmadi is with the Department of Biomedical Engineering at the University of Houston, Houston, TX 77004 USA (e-mail: mbadri-ahmadi@uh.edu). Sandipan Pati is with the Department of Neurology at McGovern Medical School, University of Texas Health and Science Center, Houston, TX 77030 (e-mail: Sandipan.Pati@uth.tmc.edu). Joseph T. Francis is with the Department of Biomedical Engineering and joint with the Department of Electrical and Computer Engineering at the University of Houston, Houston, TX 77004 USA (e-mail: jtfranci@uh.edu).
This work was supported in part by NSF grants 1942585 - CAREER: MINDWATCH: Multimodal Intelligent Noninvasive brain state Decoder for Wearable AdapTive Closed-loop arcHitectures, 1755780 – CRII: CPS: Wearable-Machine Interface Architectures to RTF, and 1527558 – NRI: Collaborative Research: Multimodal Brain Computer Interface for Human-Robot Interaction, NIH grant 1R01NS092894-01 – Towards an Autonomous Brain Machine Interface: Integrating Sensorimotor Reward Modulation and Reinforcement Learning to JTF, and NSF IUCRC BRAIN award 1650536 to JLC. Rose T. Faghih served as the senior author.

$$J_{k,n} = \sigma^2_{k,n|k,n}(\sigma^2_{k+1,n|k,n})^{-1} \quad (8)$$

$$\sigma^2_{k,n|K,n} = \sigma^2_{k,n|k,n} + J^2_{k,n}(\sigma^2_{k+1,n|k,n} - \sigma^2_{k+1,n|K,n}) \quad (9)$$

for k = K-1,...,1 and initial conditions $x_{K,n|K,n}$ and $\sigma^2_{K,n|K,n}$.

c) *State-Space Covariance Algorithm*: We then estimate the covariance $\sigma_{k,n,u,n|k,n}$ using the state-space covariance algorithm as follows [14]:

$$\sigma_{k,n,u,n|k,n} = J_{k,n}\sigma_{k+1,n,u,n|k,n} \quad (10)$$

for $1 \le k \le u \le K$.
Next, the state variance and covariance terms, $W^2_{k,n|K,n}$ and $W_{k-1,n,k,n|K,n}$ are computed where [14]:

$$W^2_{k,n|K,n} = \sigma^2_{k,n|K,n} + x^2_{k,n|K,n} \quad (11)$$

$$W_{k-1,n,k,n|K,n} = \sigma_{k-1,n,k,n|K,n} + x_{k-1,n|K,n}x_{k,n|K,n} \quad (12)$$

2) *Maximization Step*: The expectation of the log likelihood of the data is then maximized with respect to $\theta_n^{(l+1)}$ as follows [11]:

$$\rho_n^{(l+1)} = \sum_{k=1}^{K} W_{k-1,k,n|K,n}[\sum_{k=1}^{K} W_{k-1,n|K,n}]^{-1} \quad (13)$$

$$x_{0,n}^{(l+1)} = \rho_n x_{1,n|k,n} \quad (14)$$

$$\sigma_{\epsilon,n}^{2(l+1)} = K^{-1}\sum_{k=1}^{K} v^2_{k,n} + K\alpha_n^{2(l+1)} + \beta_n^{2(l+1)}\sum_{k=1}^{K} W^2_{k,n|K,n} - 2\alpha_n^{(l+1)}\sum_{k=1}^{K} v_{k,n} - 2\beta_n^{(l+1)}\sum_{k=1}^{K} x_{k,n|K,n}v_{k,n} + 2\alpha_n^{(l+1)}\beta_n^{(l+1)}\sum_{k=1}^{K} x_{k,n|K,n} \quad (15)$$

$$\begin{bmatrix}\alpha_n^{(l+1)} \\ \beta_n^{(l+1)}\end{bmatrix} = \begin{bmatrix} K & \sum_{k=1}^{K} x_{k,n|K,n} \\ \sum_{k=1}^{K} x_{k,n|K,n} & \sum_{k=1}^{K} W^2_{k,n|K,n}\end{bmatrix}^{-1} \begin{bmatrix}\sum_{k=1}^{K} v_{k,n} \\ \sum_{k=1}^{K} x_{k,n|K,n}v_{k,n}\end{bmatrix} \quad (16)$$

$$\sigma_{\eta,n}^{2(l+1)} = K^{-1}\sum_{k=1}^{K}[W^2_{k,n|K,n} - 2\rho_n^{(l+1)}W_{k-1,k,n|K,n} + \rho_n^{2(l+1)}W_{k-1,n|K,n}] \quad (17)$$

II. Kalman Filter

As the mixed filter employs a single continuous feature and a single binary feature to return a single estimation, the resulting matrix is now composed of n rows. When $n > 1$, the Kalman filter takes the multiple estimations and produces a combined estimated seizure state. The combined seizure estimation model is defined with a linear state-space framework such that z_k is a hidden combined seizure state at time $k = 1, 2, ..., K$:

$$z_k = Az_{k-1} + \omega_k \quad (18)$$

$$\boldsymbol{x}_k = \boldsymbol{B} + \boldsymbol{C}z_k + \boldsymbol{\nu}_k \quad (19)$$

where A is the unknown scalar state transition. $\boldsymbol{B}$ is the unknown bias vector of N length, where N is the total number of estimations and $\boldsymbol{B}(n)$ is the mean of the $n-th$ estimation. $\boldsymbol{C}$ is the unknown measurement transition vector, which estimates the variance for each input. $\omega_k \sim N(0, \Sigma^2_\omega)$ and $\boldsymbol{\nu}_k \sim N(0, \boldsymbol{\Sigma}^2_\nu)$ are independent unknown noise variance matrices with Σ_ω and $\boldsymbol{\Sigma}_\nu$ unknown covariances associated with the process and measurements noises, respectively.

The Kalman filter consists of two steps. The first is a prediction step, including a state mean prediction, $\bar{z}_{k|k-1}$, and a prediction of the state variance, $\Sigma^2_{k|k-1}$, as:

$$\bar{z}_{k|k-1} = A\bar{z}_{k-1|k-1} \quad (20)$$

$$\Sigma^2_{k|k-1} = A\Sigma^2{}_{k-1|k-1}A' + \Sigma_\omega. \quad (21)$$

The second step is an update step, where the filter is updated to provide a better prediction of the system:

$$\bar{z}_{k|k} = \bar{z}_{k|k-1} + L_k(\boldsymbol{x}_k - \boldsymbol{C}\bar{z}_{k|k-1} - \boldsymbol{B}) \quad (22)$$

$$\boldsymbol{\Sigma}_{k|k} = \Sigma^2_{k|k-1} - L_k(\boldsymbol{C}\Sigma^2_{k|k-1}\boldsymbol{C}' + \boldsymbol{\Sigma}_\nu)L'_k \quad (23)$$

$$L_k = \Sigma^2_{k|k-1}\boldsymbol{C}'(\boldsymbol{C}\Sigma^2_{k|k-1}\boldsymbol{C}' + \boldsymbol{\Sigma}_\nu)^{-1} \quad (24)$$

where $\boldsymbol{\Sigma}_{k|k}$ is the error covariance matrix of the state estimate of z_k given $x_k, x_{k-1}, ..., x_1$ and the the scalar L_k is the Kalman gain.

III. Square-root Kalman Filter

The formulation of the Kalman filter does not provide the numerical stability needed for this application [15], [16]. For this reason, an implementation of a square-root covariance filter algorithm is required [16] to ensure that the covariance matrix, $\boldsymbol{\Sigma}_k$, will always yield a symmetric non-negative matrix that is well conditioned. $\mathbf{S}$ is a matrix such that,

$$\boldsymbol{\Sigma}_k = \mathbf{S}_k\mathbf{S}'_k \quad (25)$$

Factoring the information matrix $\boldsymbol{\Sigma}_k^{-1}$ provides the square-root information for the filter algorithm. The prediction step from (21) can then be replaced such that

$$\begin{bmatrix}\mathbf{M}\\0\end{bmatrix} = \mathbf{G}\begin{bmatrix}\mathbf{S}'_{k+1}A'\\(\sqrt{\boldsymbol{\Sigma}_\nu})'\end{bmatrix} \tag{26}$$

where $\mathbf{M}$ is an upper triangular matrix. The matrix $\mathbf{G}$ can be constructed with the Givens transform [15], [17], [18]. Taking into account the orthogonality of matrix $\mathbf{G}$, multiplication of the transpose of (26) with itself produces:

$$\mathbf{M}'\mathbf{M} = A'\mathbf{S}_{k+1}\mathbf{S}'_{k+1}A' + \sqrt{\boldsymbol{\Sigma}_\nu}\left(\sqrt{\boldsymbol{\Sigma}_\nu}\right)' = A\Sigma_{k+1}A' + \Sigma_\omega \tag{27}$$

From (21), $\mathbf{M}$ can be substituted for $\Sigma_{k|k+1}$. Likewise, the orthogonal matrix $\mathbf{G}^*$, where

$$\begin{bmatrix}(\boldsymbol{\Sigma}_\nu + \boldsymbol{C}\Sigma_{k|k+1}\boldsymbol{C}') & L'_k\\0 & \mathbf{M}^*\end{bmatrix} = \mathbf{G}\begin{bmatrix}(\sqrt{\boldsymbol{\Sigma}_\nu})' & 0\\\mathbf{S}'_{k|k+1}\boldsymbol{C}' & \mathbf{S}'_{k|k+1}\end{bmatrix} \tag{28}$$

provides a block $\mathbf{M}^*$ in the left-hand side of (28) that can be used as $\mathbf{S}$. This is now used within the measurement update step [19], [20].

IV. Binary Seizure Estimation

The three performance criteria, sensitivity, accuracy, and specificity, are based on True Positives (TP), True Negatives (TN), False Positives (FP), False Negatives (FN), and the False Positive Rate (FPR) such that:

$$Sensitivity = \frac{TP}{TP + FN} \cdot 100\% \tag{29}$$

$$Accuracy = \frac{TP + TN}{TP + FN + TN + FP} \cdot 100\% \tag{30}$$

$$Specificity = \frac{TN}{TN + FP} \cdot 100\% = 100 - FPR \tag{31}$$

V. Combined Seizure State Estimation Plots

This section contains one combined seizure state estimation selected at random per subject with the exception of subject 08 which can be found in the main document. The figure captions contain the subject number and session for the seizure data shown (Figures 1-9). In each case the combined estimation provided a less variable prediction when compared to the best single estimation. The exception to this is Subject 6 (Figure 6), which the data had no correlation between the estimation and the actual seizure. This may be due to noise or artifacts in the recorded signals. We believe this prediction could be improved significantly by adding further data to the training and validation sets. In one instance, Subject 10 session 27, shown in Fig. 9 had class labels that were reversed, such that 1 represented no seizure activity and 0 was the occurrence of a seizure. This was resolved manually, but one solution so this does not occur would be to force the model to use the correct class labels instead of allowing the model to determine the labels without supervision.

VI. Subject 10 Feature Selection Plots

This section contains the Subject 10 individual features selected for the training set, validation set, and first seizure of the test set. The binary prediction plots are shown prior to the binarization of the data using an LDA classifier. The figure captions contain the session for the seizure data shown (Figures 11-39). Based on the data presented, we believe that a probable explanation for the decrease in accuracy for this subject is due to noise or artifacts in the training and validation sets. Examples of data that lead us to this conclusion can be seen in Figures 13, 14, 17, 18, 19, 24, and 29. In each case, the estimation does not correlate well with the determined period of seizure activity. We believe this highlights the importance of noise reduction/elimination in collected data specifically in the case of training and validation since this will impact testing sets.

VII. Selected Electrode Pairs

This section contains scalp topographical plots showing the sensors selected for each subject with the exception of subject 1, which can be found in the main paper. The beginning of each arrow represents the positive electrode where the arrow head is the negative electrode. Binary features are shown in yellow and continuous features are in blue. In some cases different bands for the same feature type, continuous or binary, from the same electrode pair were selected. In these cases a single arrow was used to show the connection (Figures 40-48). Table I contains the electrode pair locations and frequency bands chosen for all subjects and features.

References

[1] M. B. Ahmadi, A. Craik, H. F. Azgomi, J. T. Francis, J. L. Contreras-Vidal, and R. T. Faghih, "Real-time seizure state tracking using two channels: A mixed-filter approach," 2019.

[2] T. K. Moon, "The expectation-maximization algorithm," *IEEE Signal processing magazine*, vol. 13, no. 6, pp. 47–60, 1996.

[3] C. B. Do and S. Batzoglou, "What is the expectation maximization algorithm?," *Nature biotechnology*, vol. 26, no. 8, pp. 897–899, 2008.

[4] D. S. Wickramasuriya and R. T. Faghih, "A novel filter for tracking real-world cognitive stress using multi-time-scale point process observations," in *2019 41st Annual International Conference of the IEEE Engineering in Medicine and Biology Society (EMBC)*, pp. 599–602, IEEE, 2019.

[5] D. S. Wickramasuriya and R. T. Faghih, "A bayesian filtering approach for tracking arousal from binary and continuous skin conductance features," *IEEE Transactions on Biomedical Engineering*, 2019.

[6] S. Borman, "The expectation maximization algorithm-a short tutorial," *Submitted for publication*, vol. 41, 2004.

[7] G. J. McLachlan and T. Krishnan, *The EM algorithm and extensions*, vol. 382. John Wiley & Sons, 2007.

[8] D. Dreano, P. Tandeo, M. Pulido, B. Ait-El-Fquih, T. Chonavel, and I. Hoteit, "Estimating model-error covariances in nonlinear state-space models using kalman smoothing and the expectation–maximization algorithm," *Quarterly Journal of the Royal Meteorological Society*, vol. 143, no. 705, pp. 1877–1885, 2017.

[9] L. Ralaivola and F. d'Alché Buc, "Time series filtering, smoothing and learning using the kernel kalman filter," in *Proceedings. 2005 IEEE International Joint Conference on Neural Networks, 2005.*, vol. 3, pp. 1449–1454, IEEE, 2005.

[10] M. E. Khan and D. N. Dutt, "An expectation-maximization algorithm based kalman smoother approach for event-related desynchronization (erd) estimation from eeg," *IEEE transactions on biomedical engineering*, vol. 54, no. 7, pp. 1191–1198, 2007.

[11] M. J. Prerau, A. C. Smith, U. T. Eden, Y. Kubota, M. Yanike, W. Suzuki, A. M. Graybiel, and E. N. Brown, "Characterizing learning by simultaneous analysis of continuous and binary measures of performance," *Journal of neurophysiology*, vol. 102, no. 5, pp. 3060–3072, 2009.

[12] M. J. Prerau, A. C. Smith, U. T. Eden, M. Yanike, W. A. Suzuki, and E. N. Brown, "A mixed filter algorithm for cognitive state estimation from simultaneously recorded continuous and binary measures of performance," *Biological cybernetics*, vol. 99, no. 1, pp. 1–14, 2008.

[13] A. C. Smith, L. M. Frank, S. Wirth, M. Yanike, D. Hu, Y. Kubota, A. M. Graybiel, W. A. Suzuki, and E. N. Brown, "Dynamic analysis of learning in behavioral experiments," *Journal of Neuroscience*, vol. 24, no. 2, pp. 447–461, 2004.

[14] P. D. JONG and M. J. Mackinnon, "Covariances for smoothed estimates in state space models," *Biometrika*, vol. 75, no. 3, pp. 601–602, 1988.

[15] M. S. Grewal and A. P. Andrews, *Kalman filtering: Theory and Practice with MATLAB*. John Wiley & Sons, 2014.

[16] B. D. Anderson and J. B. Moore, *Optimal filtering*. Courier Corporation, 2012.

[17] C. Thornton and G. Bierman, "Givens transformation techniques for kalman filtering," *Acta Astronautica*, vol. 4, no. 7-8, pp. 847–863, 1977.

[18] R. Stewart and R. Chapman, "Fast stable kalman filter algorithms utilising the square root," in *International Conference on Acoustics, Speech, and Signal Processing*, pp. 1815–1818, IEEE, 1990.

[19] D. Simon, *Optimal state estimation: Kalman, H infinity, and nonlinear approaches*. John Wiley & Sons, 2006.

[20] F. Tusell *et al.*, "Kalman filtering in r," *Journal of Statistical Software*, vol. 39, no. 2, pp. 1–27, 2011.

TABLE I: Selected continuous and binary feature locations with the selected frequency bands for each feature and subject.

Channel and Band Selection

Continuous Feature

	One		Two		Three		Four		Five	
**Subject**	Location	Band	Location	Band	Location	Band	Location	Band	Location	Band
1	T7-FT9	Beta	C4-P4	Beta	T7-FT9	Theta	F8-T8	Alpha	P8-O2	Beta
2	T8-P8	Delta	F4-C4	Theta	F3-C3	Delta	P8-O2	Delta	T8-P8	Alpha
3	Cz-Pz	Theta	Cz-Pz	Delta	Fz-Cz	Theta	P8-O2	Theta	F3-C3	Theta
4	F4-C4	Theta	C4-P4	Delta	T8-P8	Theta	Fz-Cz	Delta	T8-P8	Delta
5	P4-O2	Alpha	FP2-F4	Beta	Cz-Pz	Beta	FP1-F3	Alpha	T7-FT9	Theta
6	F7-T7	Beta	P7-T7	Beta	P7-T7	Alpha	F7-T7	Alpha	T7-FT9	Alpha
7	F7-T7	Theta	P7-T7	Alpha	P7-T7	Beta	F7-T7	Beta	T7-FT9	Beta
8	Cz-Pz	Delta	P7-T7	Delta	P4-O2	Theta	Fz-Cz	Delta	T8-P8	Delta
9	P7-T7	Theta	C4-P4	Beta	P4-O2	Beta	P4-O2	Alpha	T7-FT9	Delta
10	T7-P7	Theta	Cz-Pz	Alpha	Cz-Pz	Beta	F3-C3	Alpha	F3-C3	Alpha

Binary Feature

	One		Two		Three		Four		Five	
**Subject**	Location	Band	Location	Band	Location	Band	Location	Band	Location	Band
1	T8-P8	Theta	P3-O1	Theta	C4-P4	Delta	P7-O1	Theta	C3-P3	Theta
2	FP2-F4	Beta	FP2-F4	Theta	P7-O1	Delta	Fz-Cz	Theta	FT9-FT10	Delta
3	P7-O1	Theta	P8-O2	Beta	P8-O2	Delta	P7-O1	Delta	P3-O1	Delta
4	Fz-Cz	Delta	P3-O1	Beta	T8-P8	Beta	C3-P3	Delta	P4-O2	Delta
5	P8-O2	Alpha	P4-O2	Theta	T8-P8	Delta	P8-O2	Theta	FP2-F4	Theta
6	P4-O2	Delta	P8-O2	Delta	T7-P7	Alpha	P7-T7	Beta	T8-P8	Delta
7	P4-O2	Delta	P8-O2	Delta	T7-P7	Alpha	P7-T7	Beta	T8-P8	Delta
8	P4-O2	Delta	T7-P7	Delta	T7-P7	Delta	P7-T7	Delta	F4-C4	Theta
9	F3-C3	Alpha	P4-O2	Alpha	T7-P7	Alpha	FP2-F4	Alpha	T7-FT9	Theta
10	F7-T7	Theta	T7-P7	Theta	F7-T7	Alpha	F3-C3	Theta	Fz-Cz	Alpha

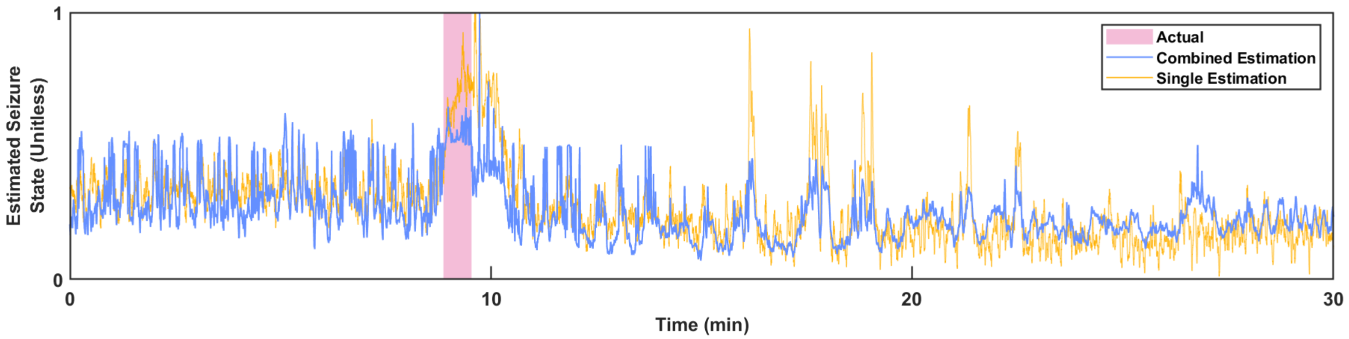

Fig. 1: **Estimated Seizure State for Subject 1, session 15.** Combined seizure state estimation and the first individual estimation from this process. The shaded area marks the period designated as the true seizure state. In this instance the combined estimation appears to be worse than the single estimation, but the single estimation has multiple large increases in seizure prediction several minutes after the seizure whereas the combined estimation does not.

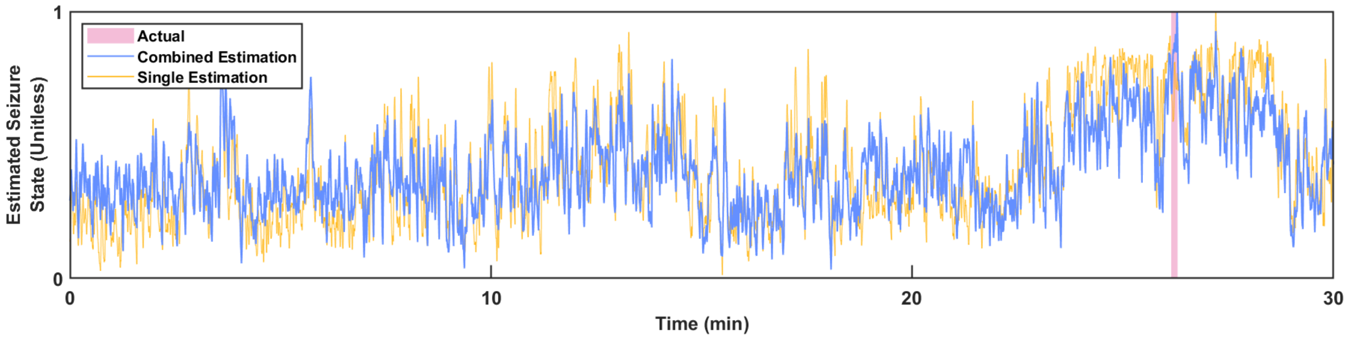

Fig. 2: **Estimated Seizure State for Subject 2, session 19.** Combined seizure state estimation and the first individual estimation from this process. The shaded area marks the period designated as the true seizure state.While the period of seizure activity for this subject and session is short, the combined estimation still correlates with the seizure activity. While the combined estimation follows a similar pattern, there is less variability in the prediction, providing a better estimation overall.

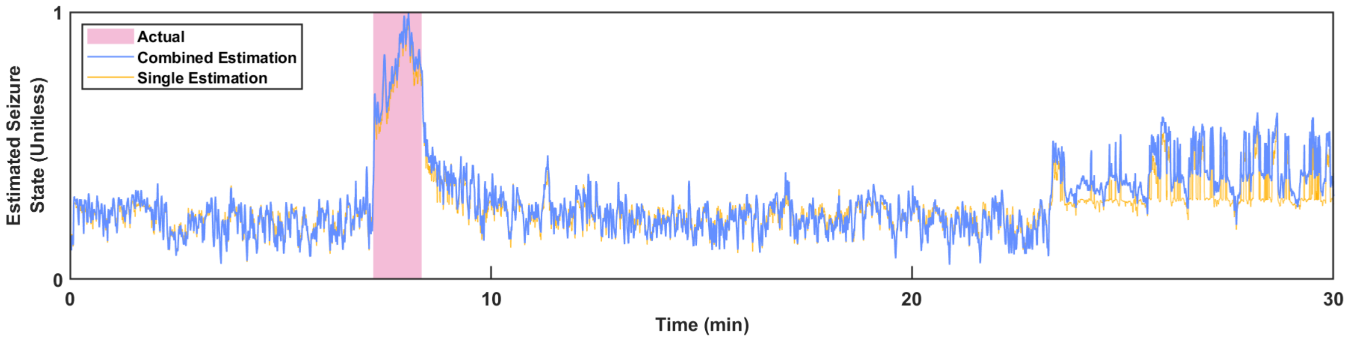

Fig. 3: **Estimated Seizure State for Subject 3, session 3.** Combined seizure state estimation and the first individual estimation from this process. The shaded area marks the period designated as the true seizure state.Here the combined estimation predicts a higher value than the single estimation during the actual seizure activity and has reduced variability.

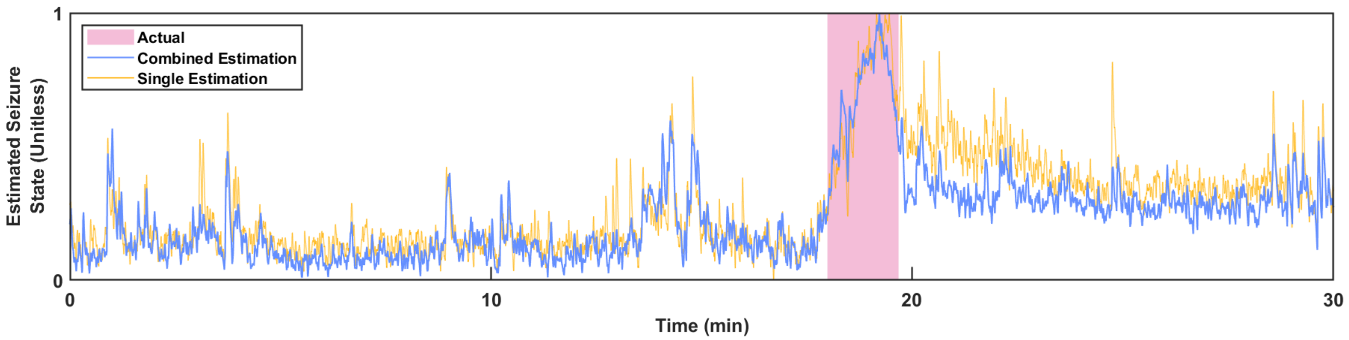

Fig. 4: **Estimated Seizure State for Subject 4, session 28.** Combined seizure state estimation and the first individual estimation from this process. The shaded area marks the period designated as the true seizure state. In this instance the single estimation has a higher prediction after the seizure, while the combined estimation drops directly after the marked period and remains lower than the single prediction.

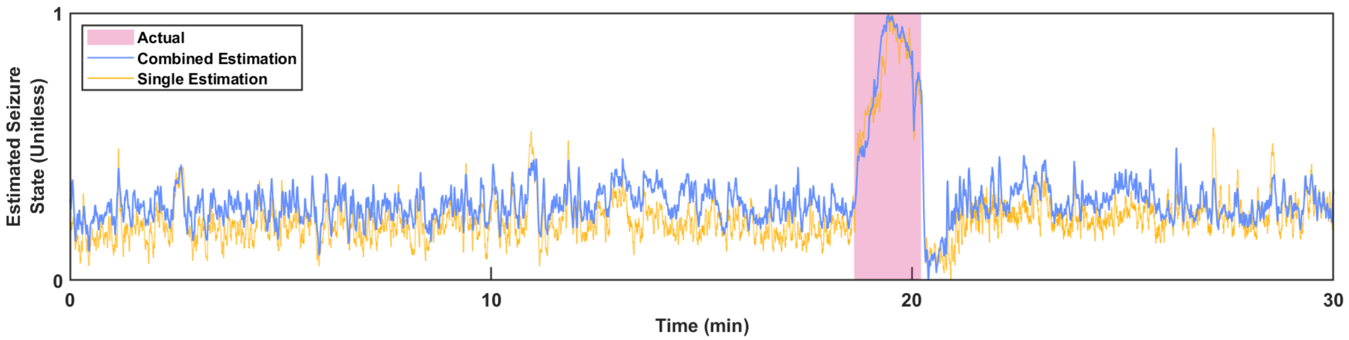

Fig. 5: **Estimated Seizure State for Subject 5, session 16.** Combined seizure state estimation and the first individual estimation from this process. The shaded area marks the period designated as the true seizure state.The combined estimation provides a clear and correct prediction with overall reduced noise when compared to the single estimation.

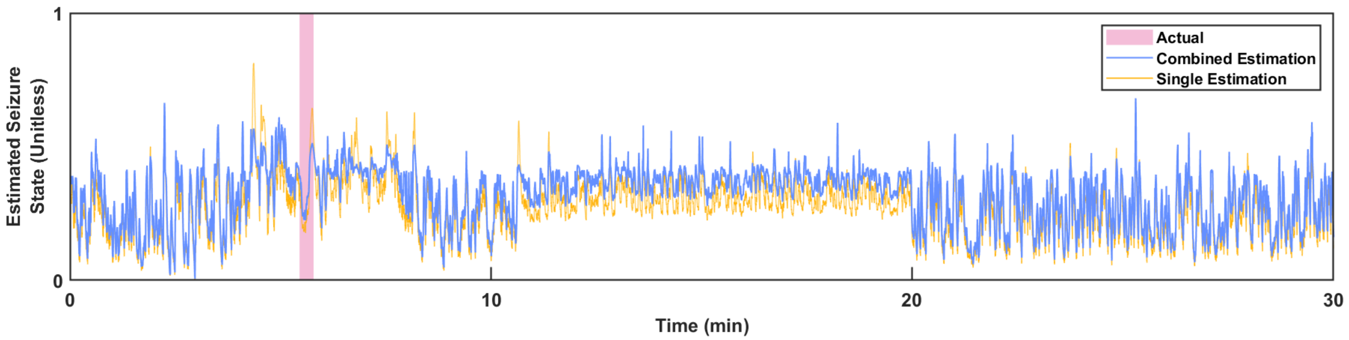

Fig. 6: **Estimated Seizure State for Subject 6, session 4.** Combined seizure state estimation and the first individual estimation from this process. The shaded area marks the period designated as the true seizure state. These data shows an instance with lack of correlation between the single estimation or combined estimation with the actual seizure, which may be due to noise and/or artifacts in the recorded signals.

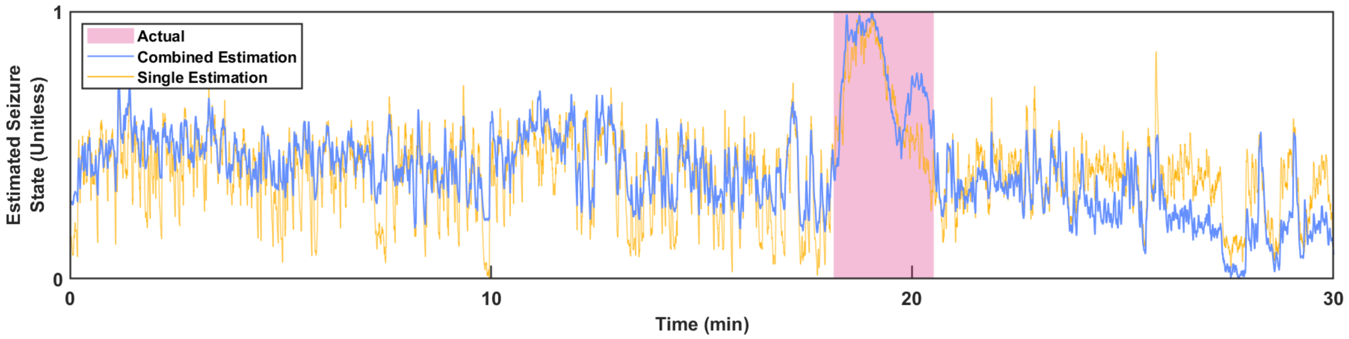

Fig. 7: **Estimated Seizure State for Subject 7, session 19.** Combined seizure state estimation and the first individual estimation from this process. The shaded area marks the period designated as the true seizure state.The single estimation fluctuates at a higher amplitude throughout the session, while the combined estimation provides a more consistent prediction and provides a larger response during the seizure that lasts the full duration, where the single estimation prediction drops about halfway through the marked seizure.

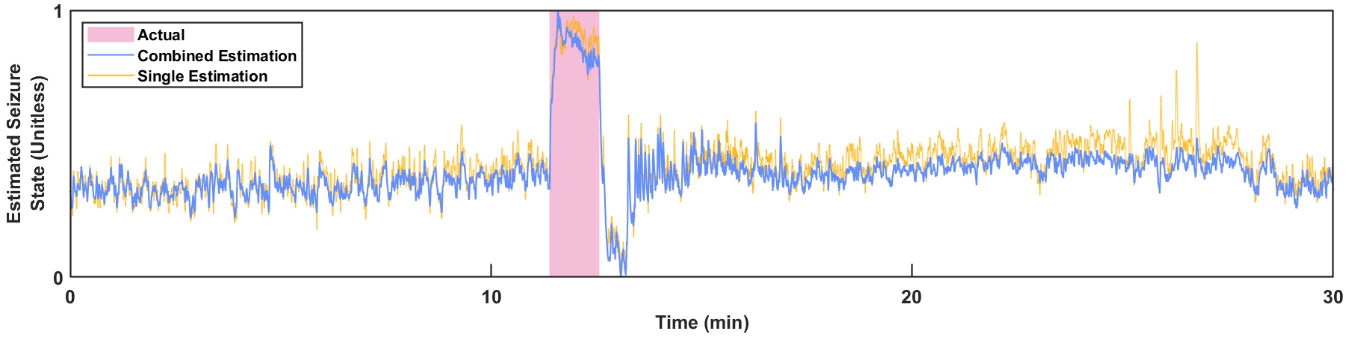

Fig. 8: **Estimated Seizure State for Subject 9, session 8.** Combined seizure state estimation and the first individual estimation from this process. The shaded area marks the period designated as the true seizure state.Both the single estimation and the combined estimation provide a clear and correct prediction for the seizure state. However the combined estimation has less variance when compared to the single estimation.

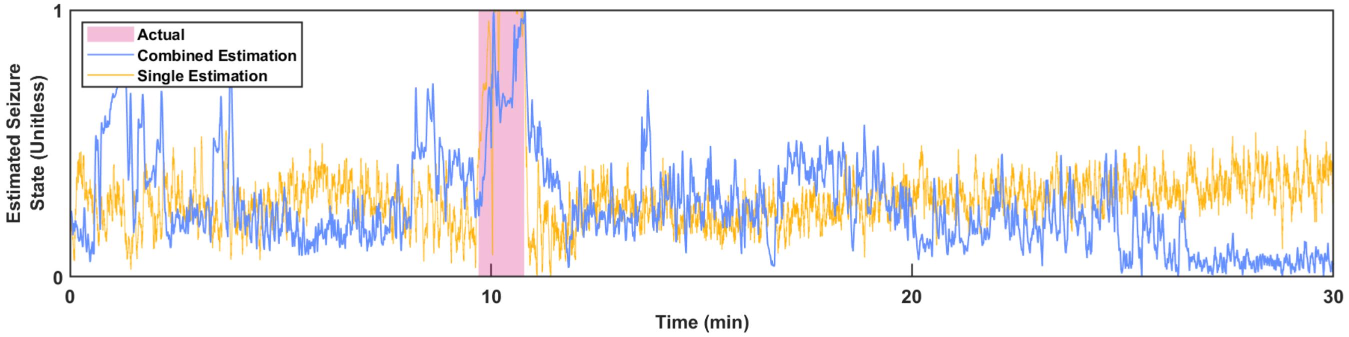

Fig. 9: **Estimated Seizure State for Subject 10, session 27.** Combined seizure state estimation and the first individual estimation from this process. The shaded area marks the period designated as the true seizure state.In this case the combined estimation has a higher variance in the prediction, but correlates well with the marked seizure activity and has a lower mean value outside the marked seizure area which provides a better estimation

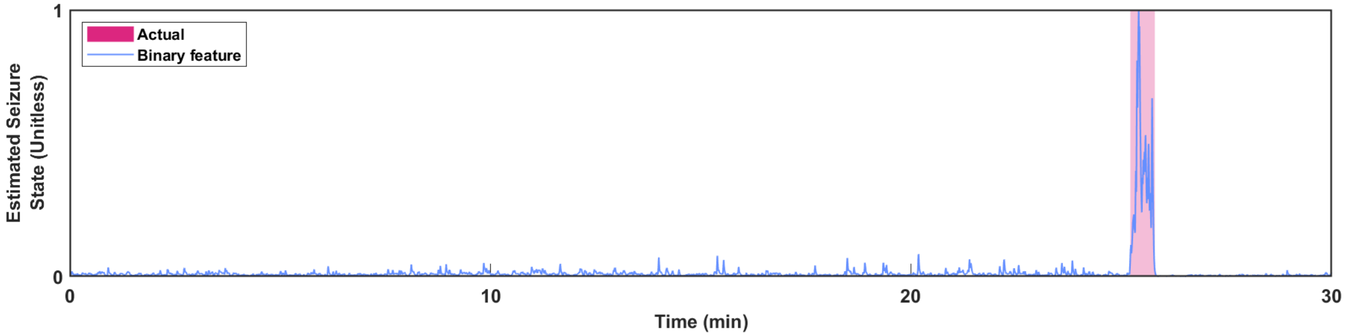

Fig. 10: **Estimated Seizure State for Subject 10, session 12.** The first binary feature selected for this session shown here prior to binarization. This session was used as the training set. The shaded area marks the period designated as the true seizure state.

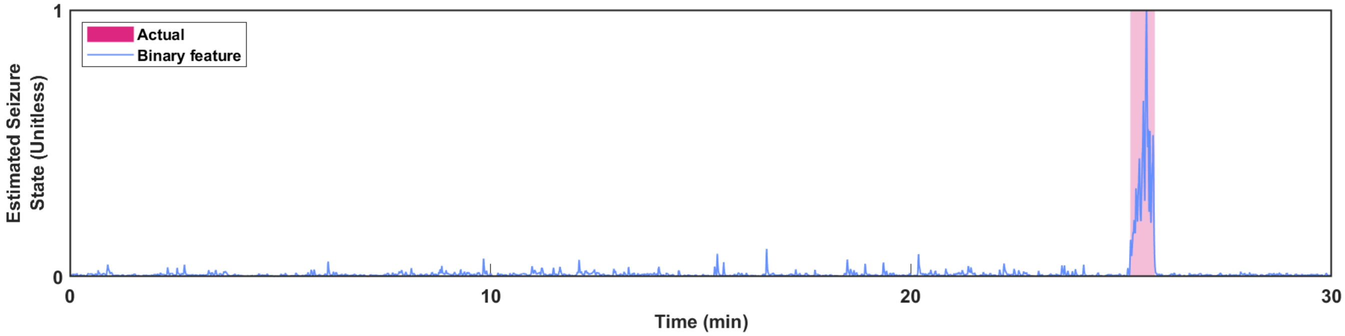

Fig. 11: **Estimated Seizure State for Subject 10, session 12.** The second binary feature selected for this session shown here prior to binarization. This session was used as the training set. The shaded area marks the period designated as the true seizure state.

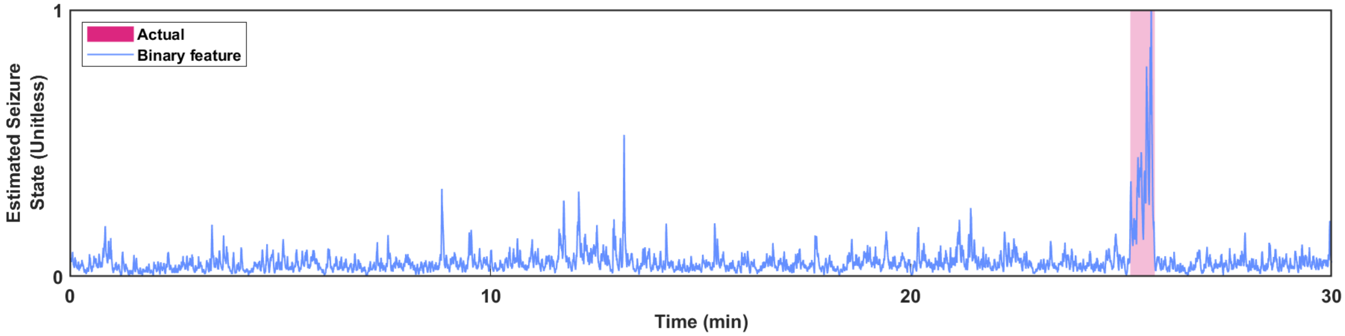

Fig. 12: **Estimated Seizure State for Subject 10, session 12.** The third binary feature selected for this session shown here prior to binarization. This session was used as the training set. The shaded area marks the period designated as the true seizure state.

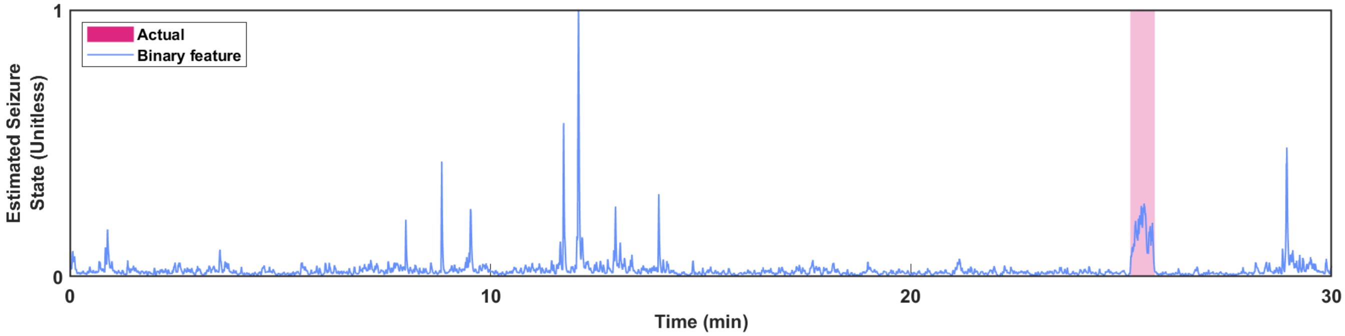

Fig. 13: **Estimated Seizure State for Subject 10, session 12.** The fourth binary feature selected for this session shown here prior to binarization. This session was used as the training set. The shaded area marks the period designated as the true seizure state.

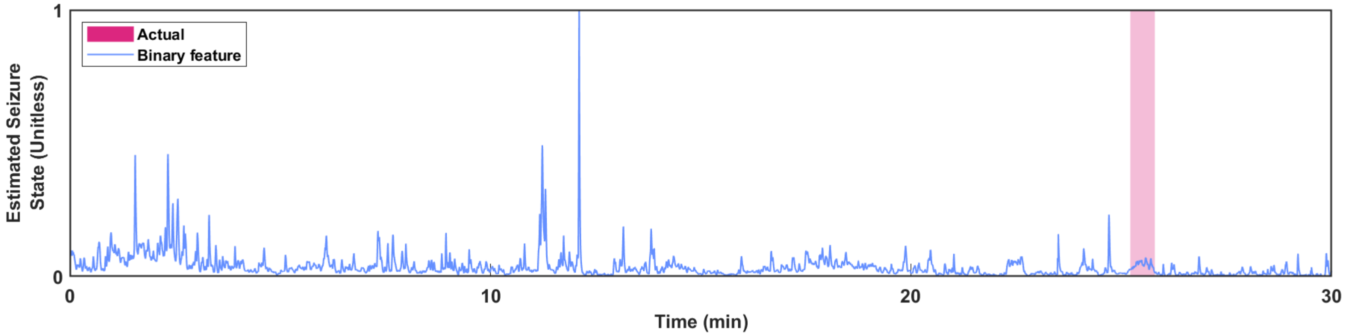

Fig. 14: **Estimated Seizure State for Subject 10, session 12.** The fifth binary feature selected for this session shown here prior to binarization. This session was used as the training set. The shaded area marks the period designated as the true seizure state.

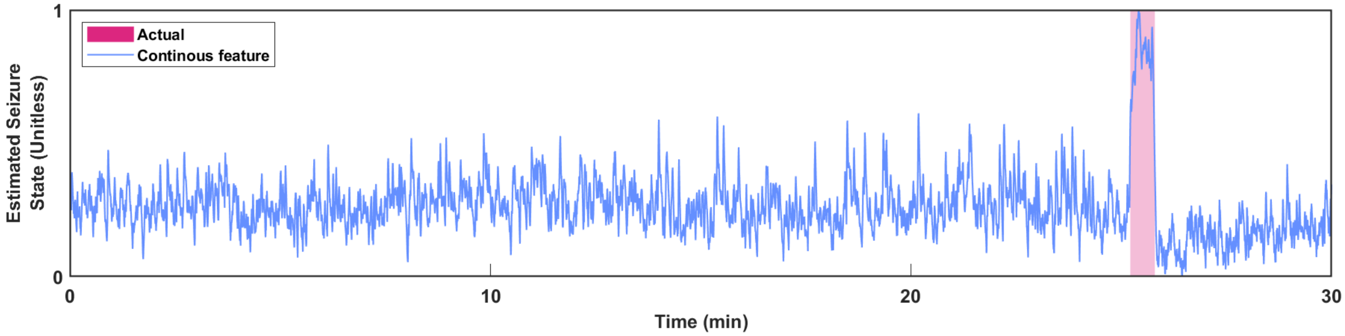

Fig. 15: **Estimated Seizure State for Subject 10, session 12.** The first continuous feature selected for this session. This session was used as the training set. The shaded area marks the period designated as the true seizure state.

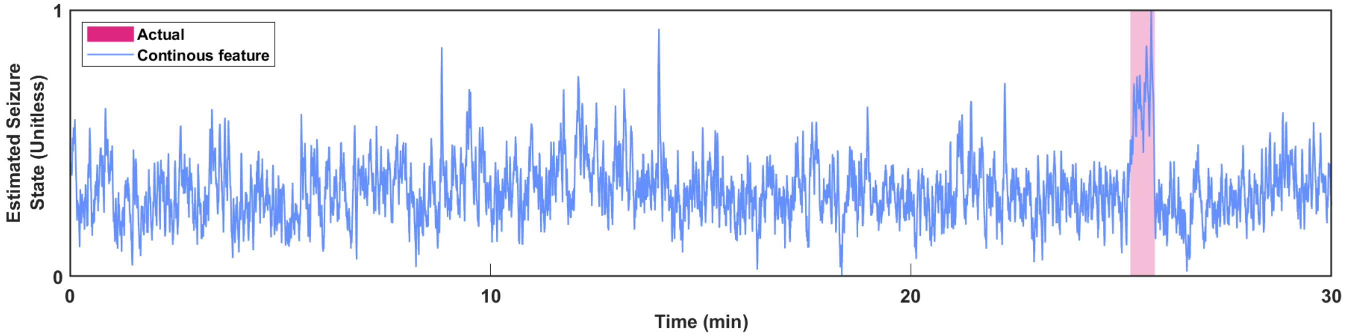

Fig. 16: **Estimated Seizure State for Subject 10, session 12.** The second continuous feature selected for this session. This session was used as the training set. The shaded area marks the period designated as the true seizure state.

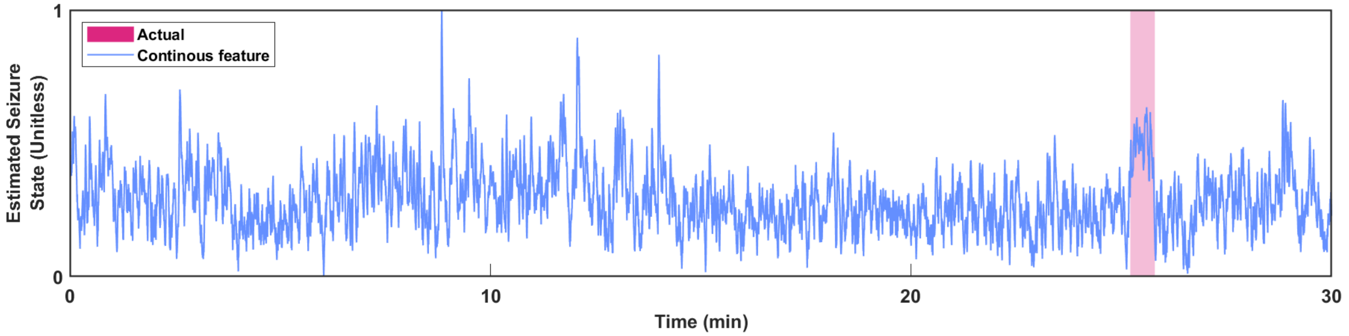

Fig. 17: **Estimated Seizure State for Subject 10, session 12.** The third continuous feature selected for this session. This session was used as the training set. The shaded area marks the period designated as the true seizure state.

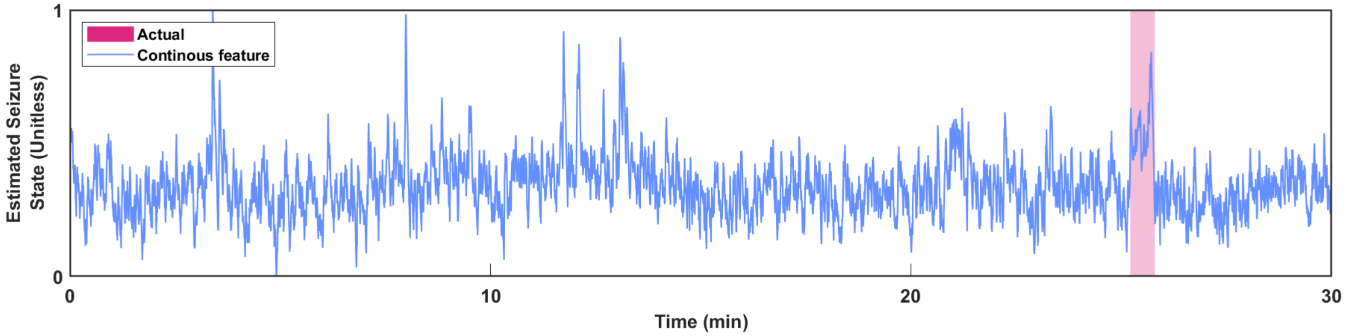

Fig. 18: **Estimated Seizure State for Subject 10, session 12.** The fourth continuous feature selected for this session. This session was used as the training set. The shaded area marks the period designated as the true seizure state.

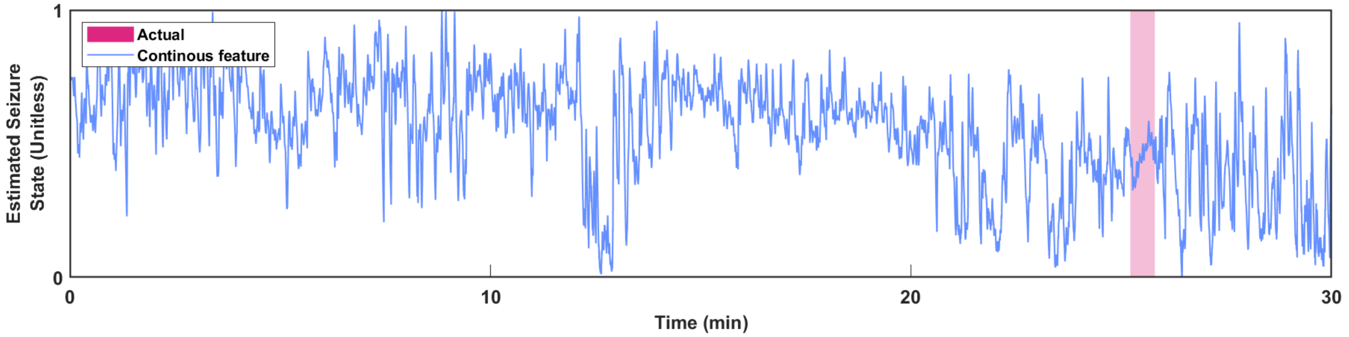

Fig. 19: **Estimated Seizure State for Subject 10, session 12.** The fifth continuous feature selected for this session . This session was used as the training set. The shaded area marks the period designated as the true seizure state.

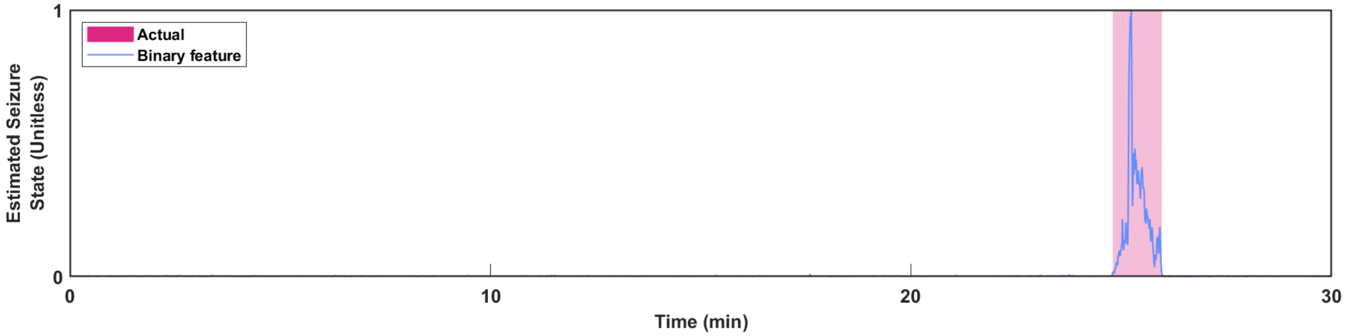

Fig. 20: **Estimated Seizure State for Subject 10, session 20.** The first binary feature selected for this session shown here prior to binarization. This session was used as the validation set. The shaded area marks the period designated as the true seizure state.

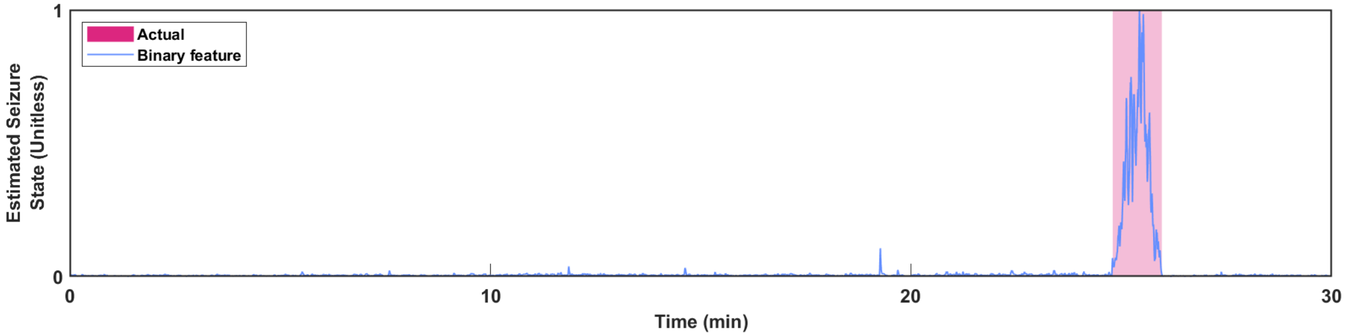

Fig. 21: **Estimated Seizure State for Subject 10, session 20.** The second binary feature selected for this session shown here prior to binarization. This session was used as the validation set. The shaded area marks the period designated as the true seizure state.

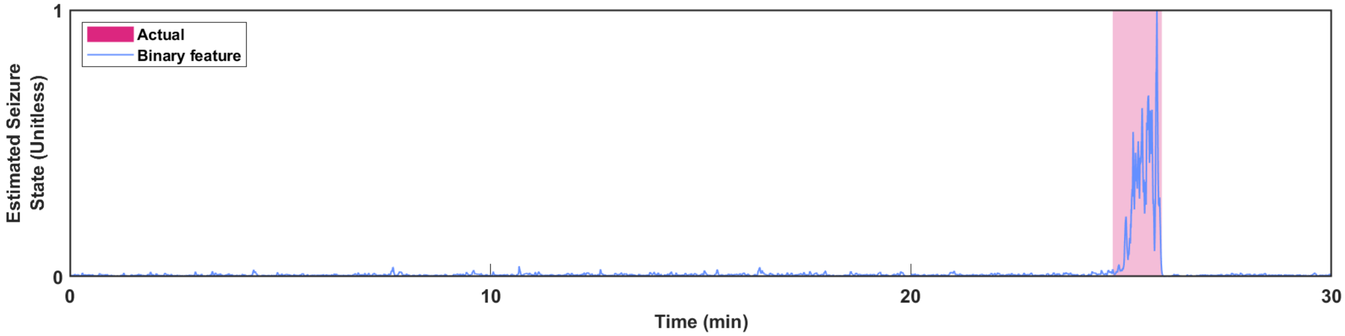

Fig. 22: **Estimated Seizure State for Subject 10, session 20.** The third binary feature selected for this session shown here prior to binarization. This session was used as the validation set. The shaded area marks the period designated as the true seizure state.

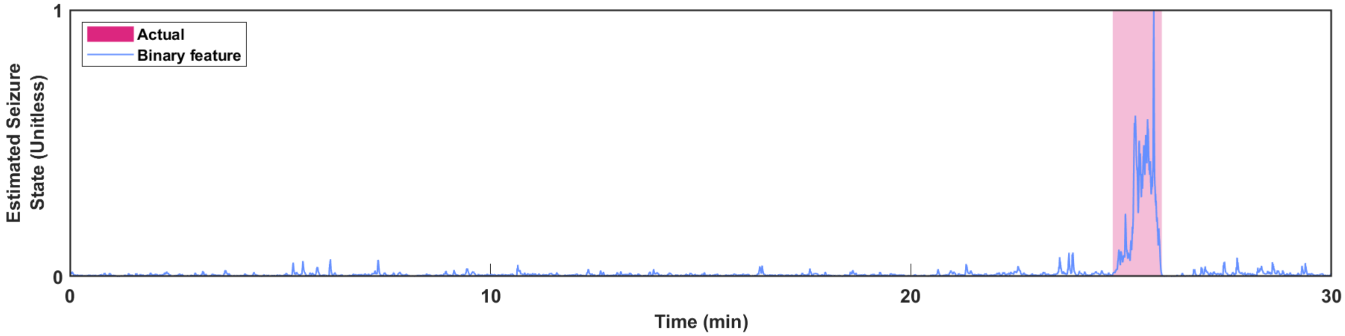

Fig. 23: **Estimated Seizure State for Subject 10, session 20.** The fourth binary feature selected for this session shown here prior to binarization. This session was used as the validation set. The shaded area marks the period designated as the true seizure state.

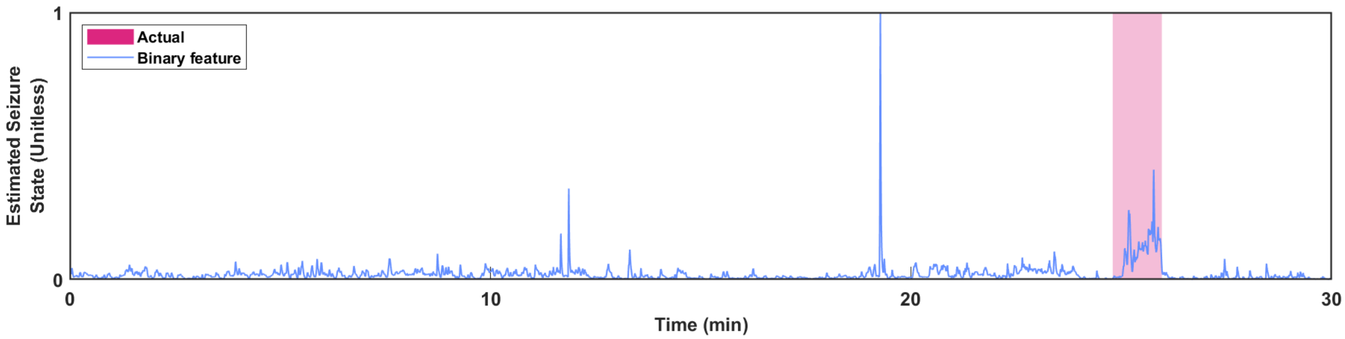

Fig. 24: **Estimated Seizure State for Subject 10, session 20.** The fifth binary feature selected for this session shown here prior to binarization. This session was used as the validation set. The shaded area marks the period designated as the true seizure state.

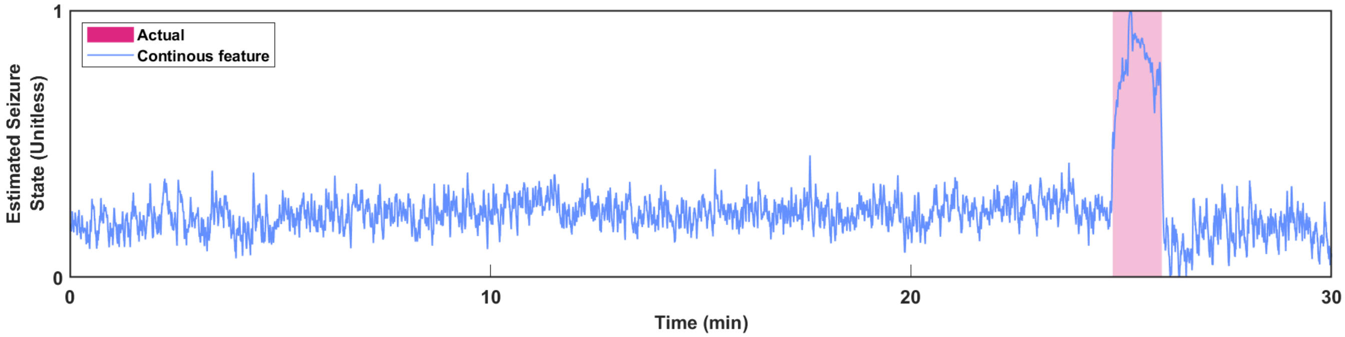

Fig. 25: **Estimated Seizure State for Subject 10, session 20.** The first continuous feature selected for this session. This session was used as the validation set. The shaded area marks the period designated as the true seizure state.

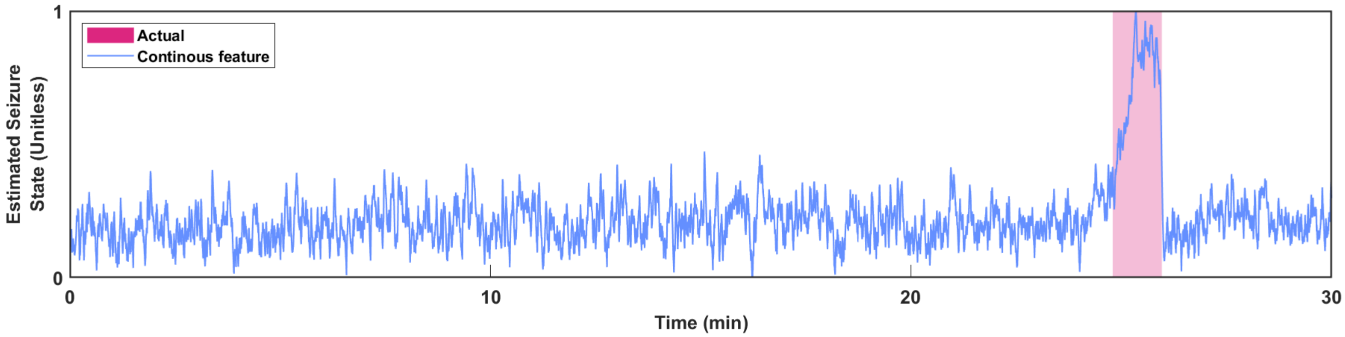

Fig. 26: **Estimated Seizure State for Subject 10, session 20.** The second continuous feature selected for this session. This session was used as the validation set. The shaded area marks the period designated as the true seizure state.

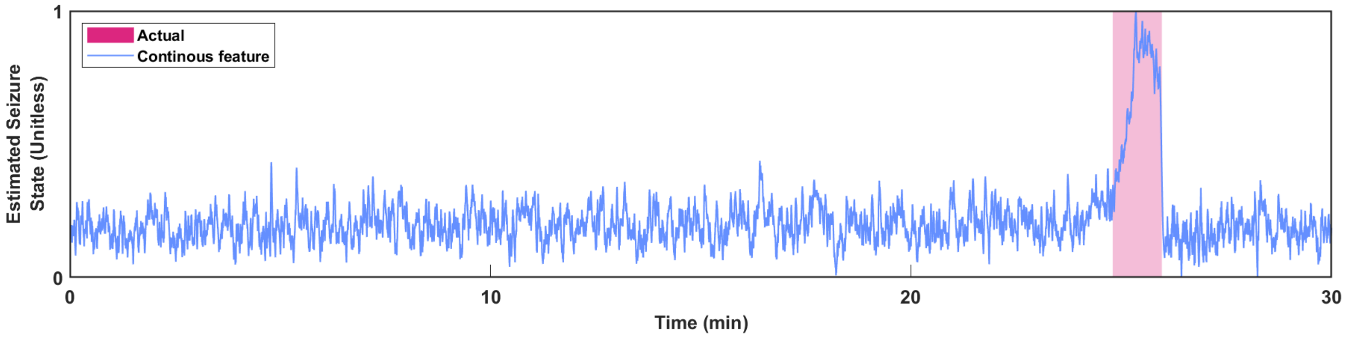

Fig. 27: **Estimated Seizure State for Subject 10, session 20.** The third continuous feature selected for this session. This session was used as the validation set. The shaded area marks the period designated as the true seizure state.

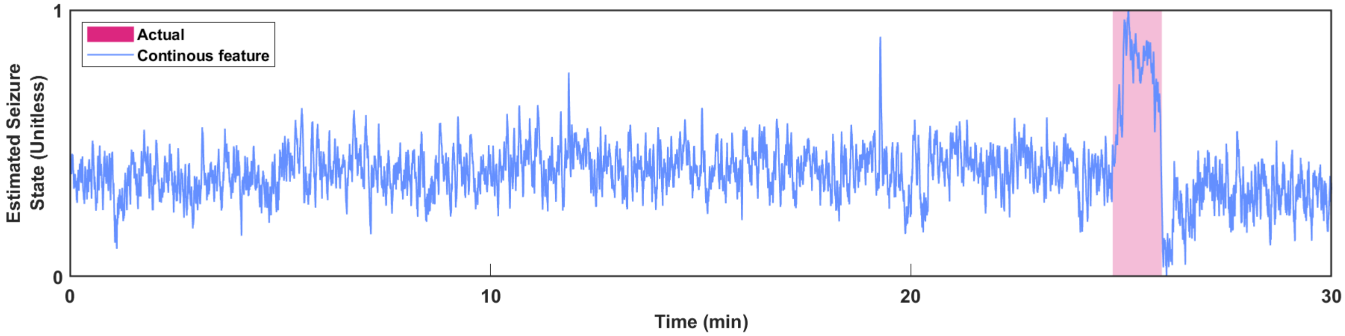

Fig. 28: **Estimated Seizure State for Subject 10, session 20.** The fourth continuous feature selected for this session. This session was used as the validation set. The shaded area marks the period designated as the true seizure state.

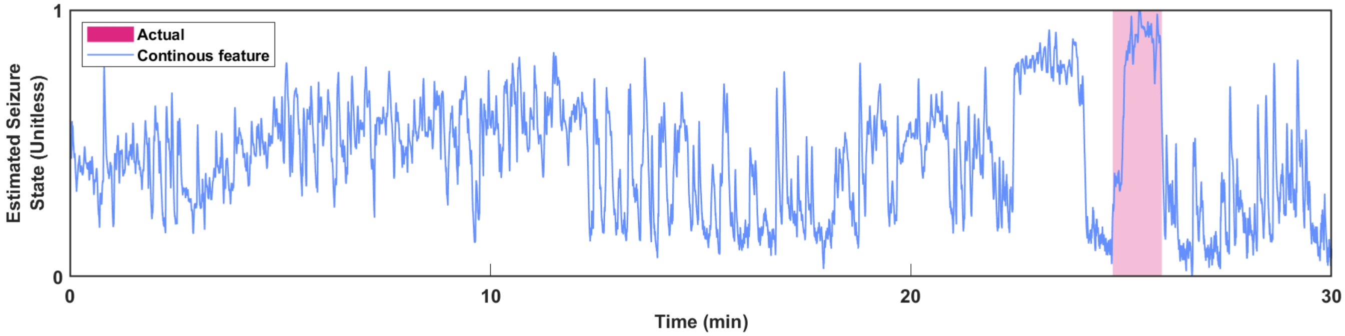

Fig. 29: **Estimated Seizure State for Subject 10, session 20.** The fifth continuous feature selected for this session. This session was used as the validation set. The shaded area marks the period designated as the true seizure state.

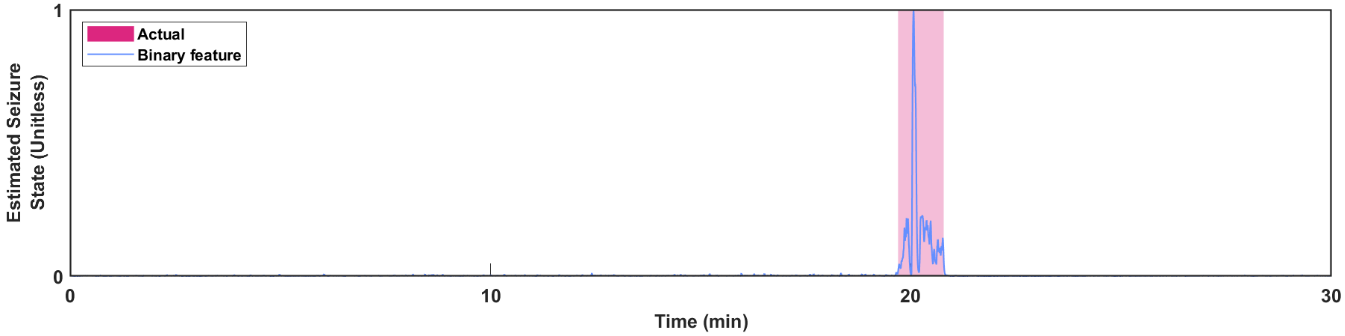

Fig. 30: **Estimated Seizure State for Subject 10, session 27.** The first binary feature selected for this session shown here prior to binarization. This session was used as part of the testing set. The shaded area marks the period designated as the true seizure state.

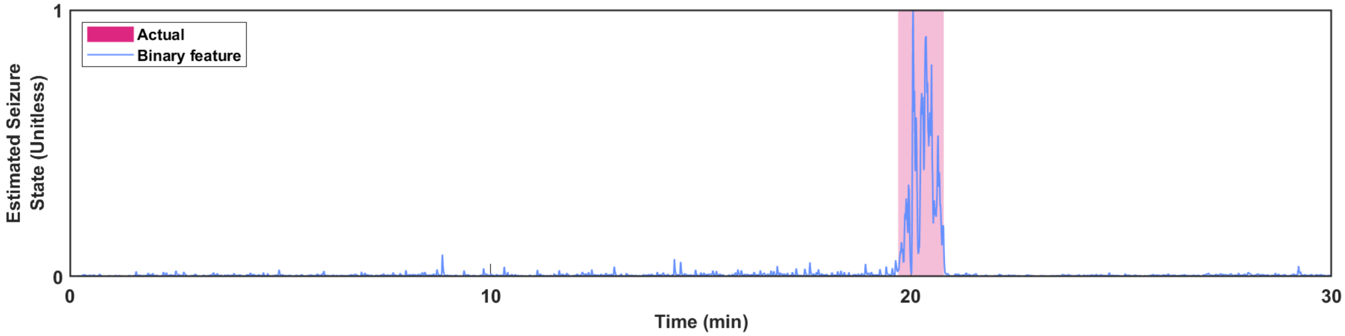

Fig. 31: **Estimated Seizure State for Subject 10, session 27.** The second binary feature selected for this session shown here prior to binarization. This session was used as part of the testing set. The shaded area marks the period designated as the true seizure state.

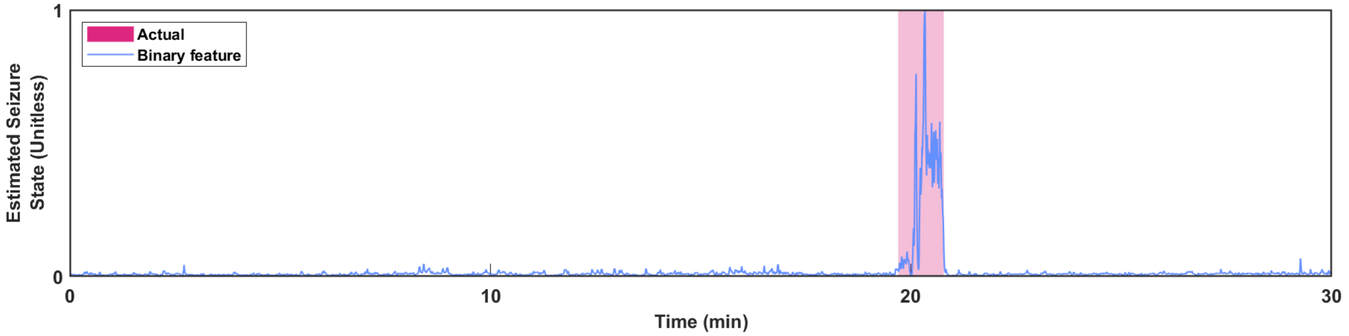

Fig. 32: **Estimated Seizure State for Subject 10, session 27.** The third binary feature selected for this session shown here prior to binarization. This session was used as part of the testing set. The shaded area marks the period designated as the true seizure state.

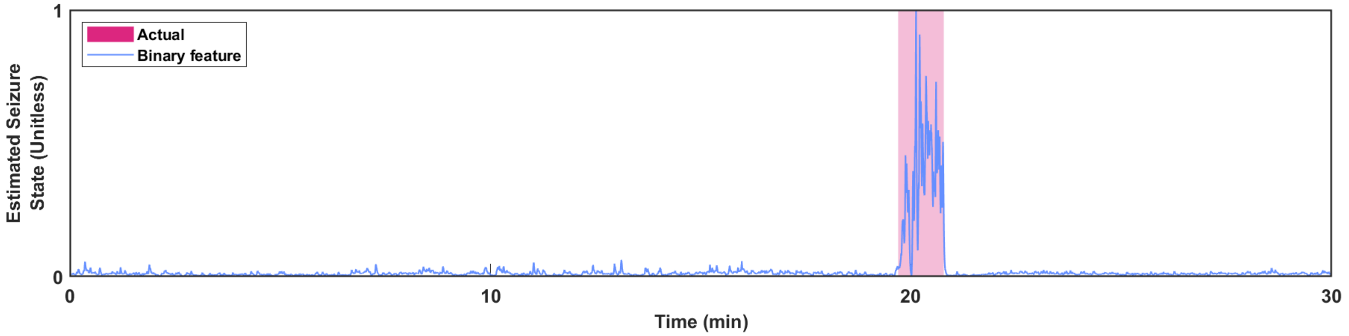

Fig. 33: **Estimated Seizure State for Subject 10, session 27.** The fourth binary feature selected for this session shown here prior to binarization. This session was used as part of the testing set. The shaded area marks the period designated as the true seizure state.

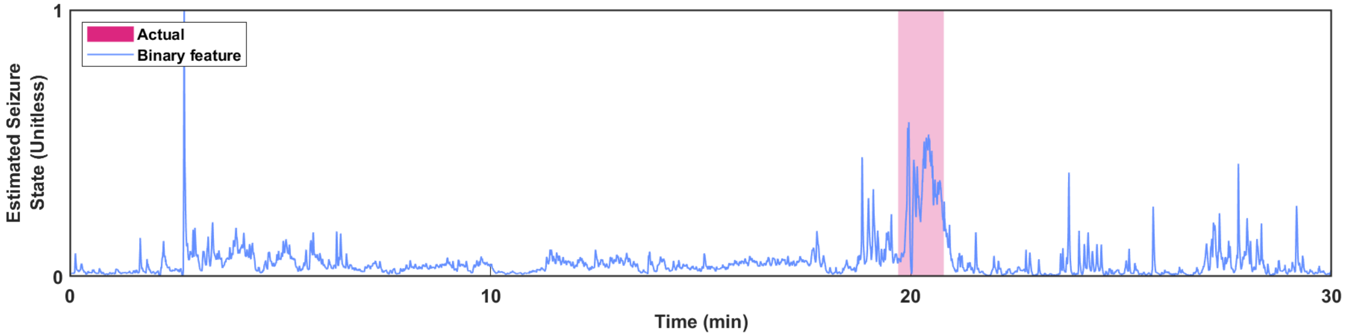

Fig. 34: **Estimated Seizure State for Subject 10, session 27.** The fifth binary feature selected for this session shown here prior to binarization. This session was used as part of the testing set. The shaded area marks the period designated as the true seizure state.

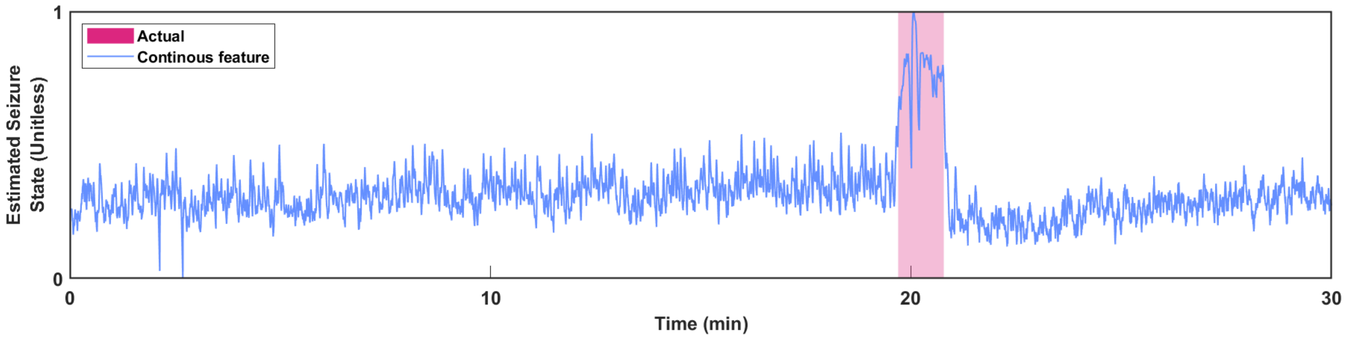

Fig. 35: **Estimated Seizure State for Subject 10, session 27.** The first continuous feature selected for this session. This session was used as part of the testing set. The shaded area marks the period designated as the true seizure state.

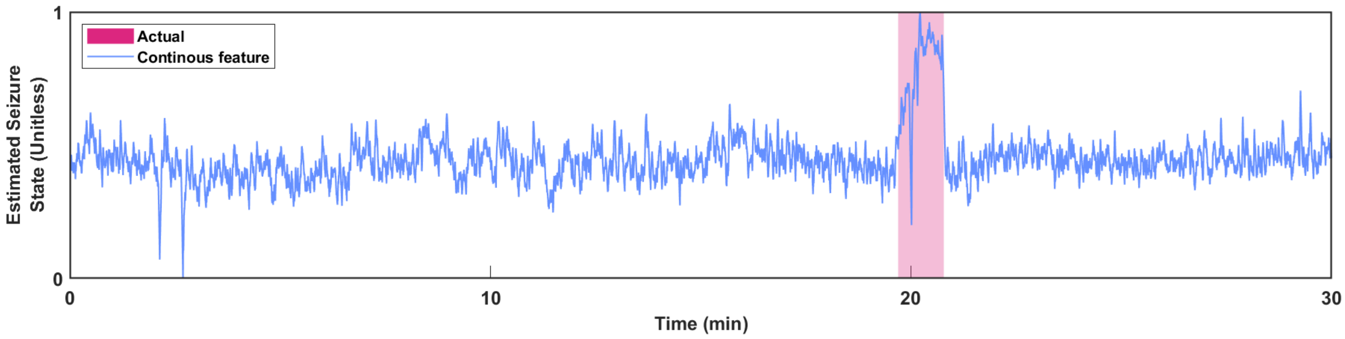

Fig. 36: **Estimated Seizure State for Subject 10, session 27.** The second continuous feature selected for this session. This session was used as part of the testing set. The shaded area marks the period designated as the true seizure state.

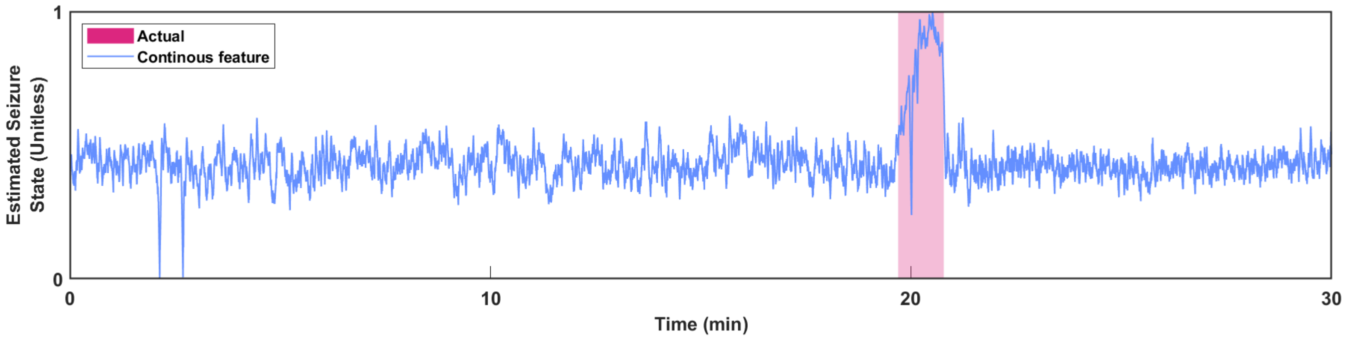

Fig. 37: **Estimated Seizure State for Subject 10, session 27.** The third continuous feature selected for this session. This session was used as part of the testing set. The shaded area marks the period designated as the true seizure state.

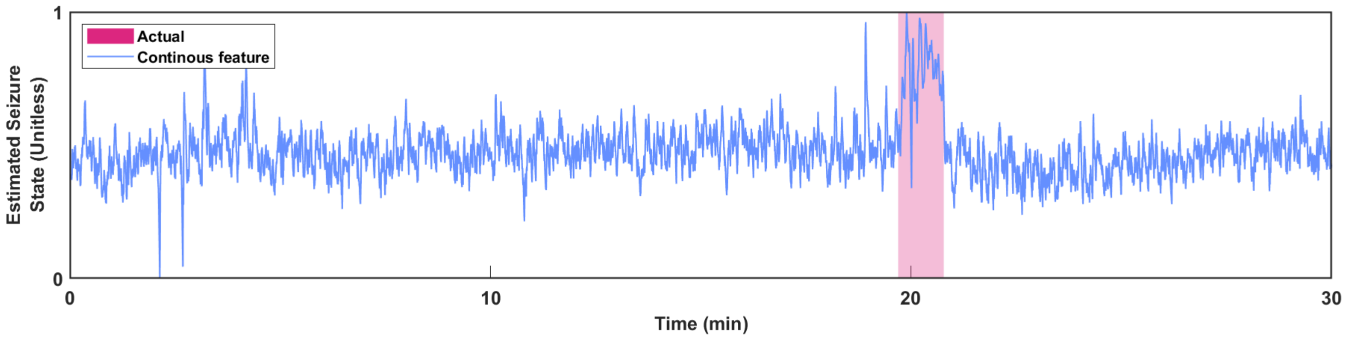

Fig. 38: **Estimated Seizure State for Subject 10, session 27.** The fourth continuous feature selected for this session. This session was used as part of the testing set. The shaded area marks the period designated as the true seizure state.

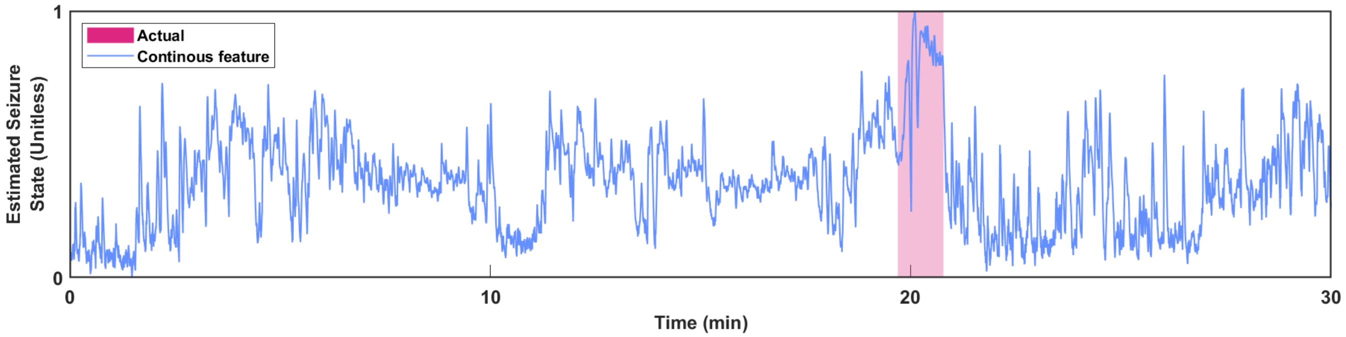

Fig. 39: **Estimated Seizure State for Subject 10, session 27.** The fifth continuous feature selected for this session. This session was used as part of the testing set. The shaded area marks the period designated as the true seizure state.

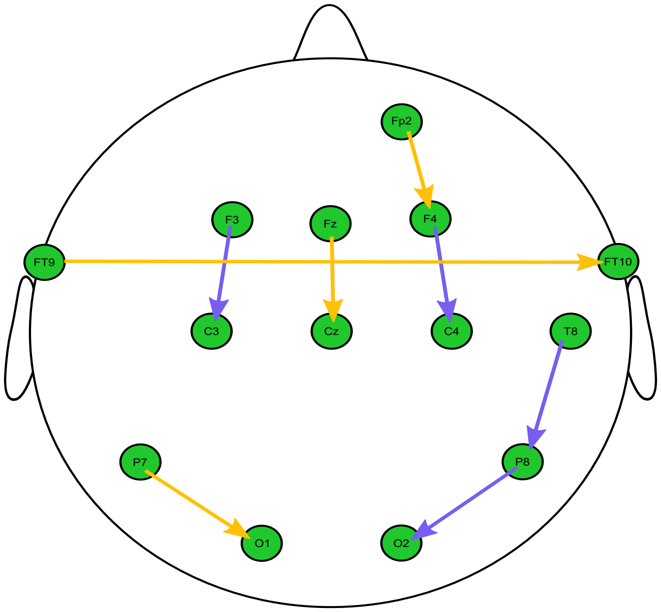

Fig. 40: **Subject 2 scalp map** Sensor pairs selected for the continuous features are shown in blue, while binary features are shown in yellow.

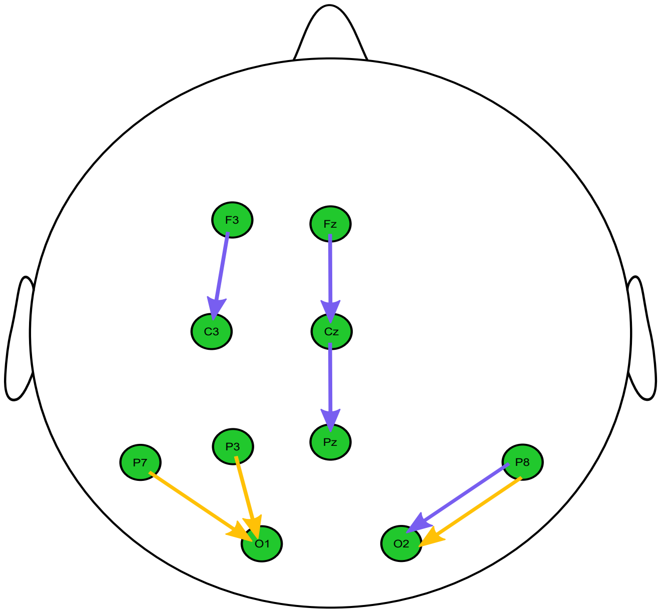

Fig. 41: **Subject 3 scalp map** Sensor pairs selected for the continuous features are shown in blue, while binary features are shown in yellow.

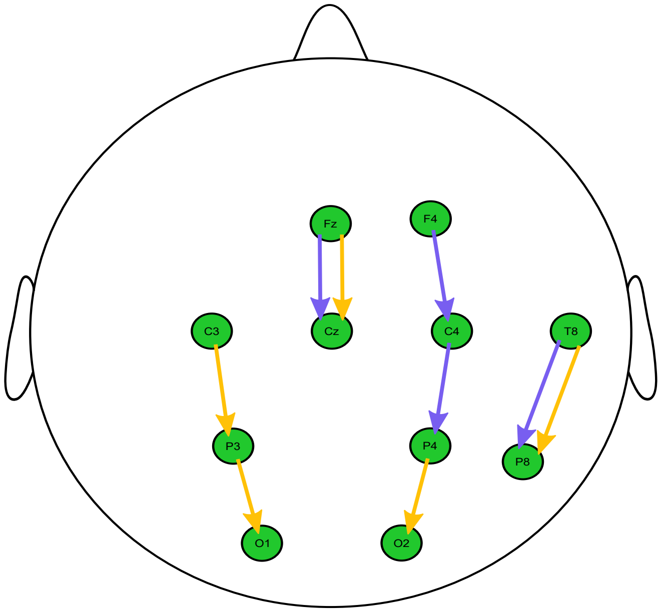

Fig. 42: **Subject 4 scalp map** Sensor pairs selected for the continuous features are shown in blue, while binary features are shown in yellow.

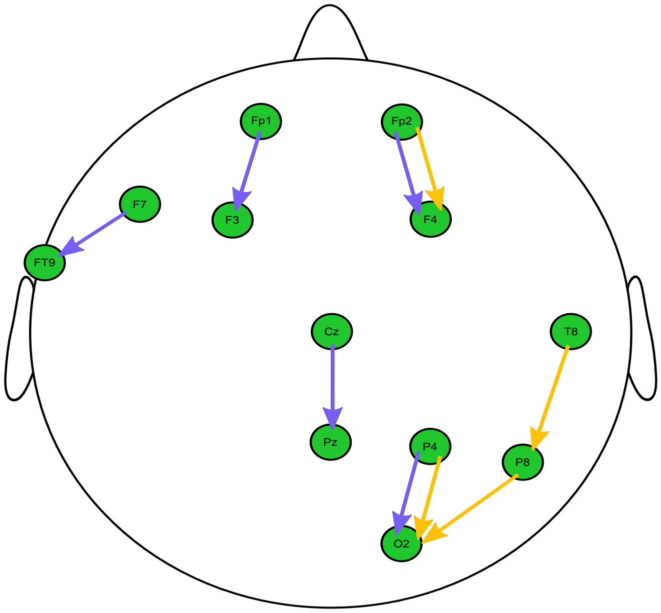

Fig. 43: **Subject 5 scalp map** Sensor pairs selected for the continuous features are shown in blue, while binary features are shown in yellow.

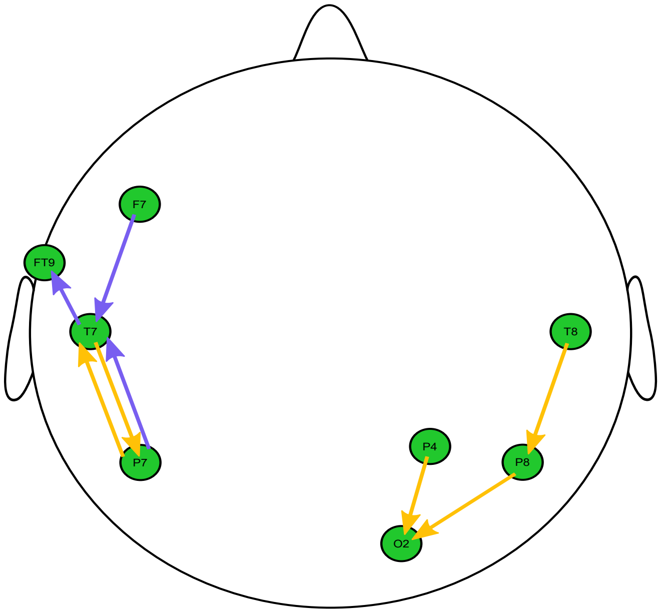

Fig. 44: **Subject 6 scalp map** Sensor pairs selected for the continuous features are shown in blue, while binary features are shown in yellow.

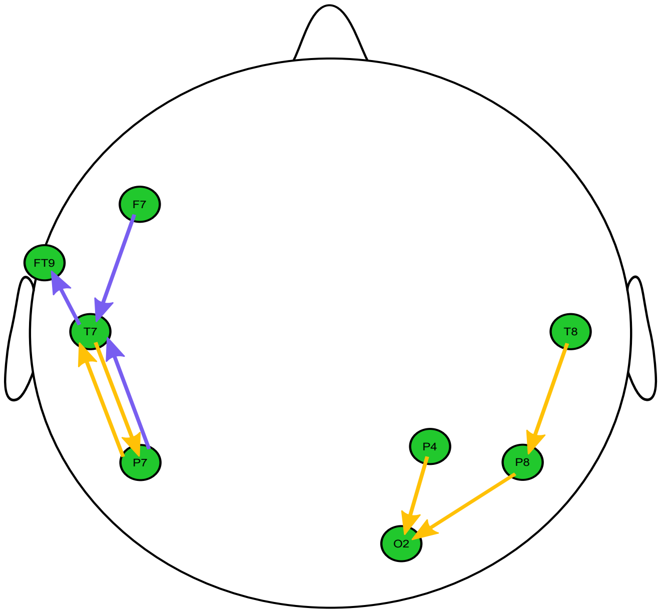

Fig. 45: **Subject 7 scalp map** Sensor pairs selected for the continuous features are shown in blue, while binary features are shown in yellow.

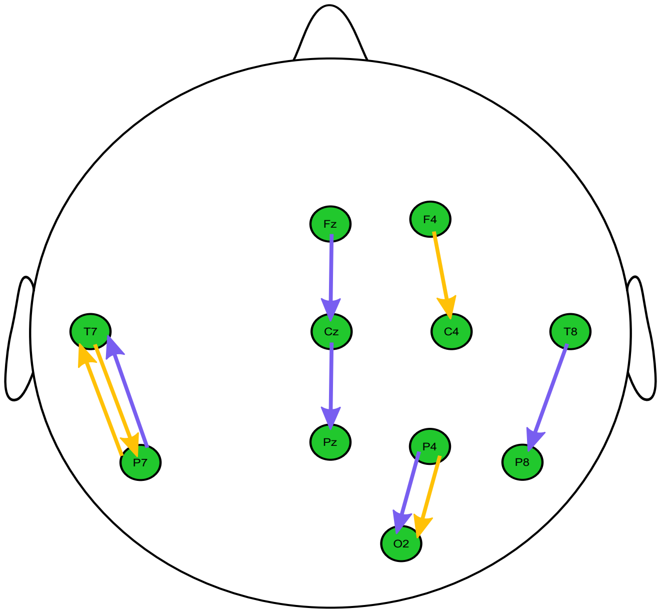

Fig. 46: **Subject 8 scalp map** Sensor pairs selected for the continuous features are shown in blue, while binary features are shown in yellow.

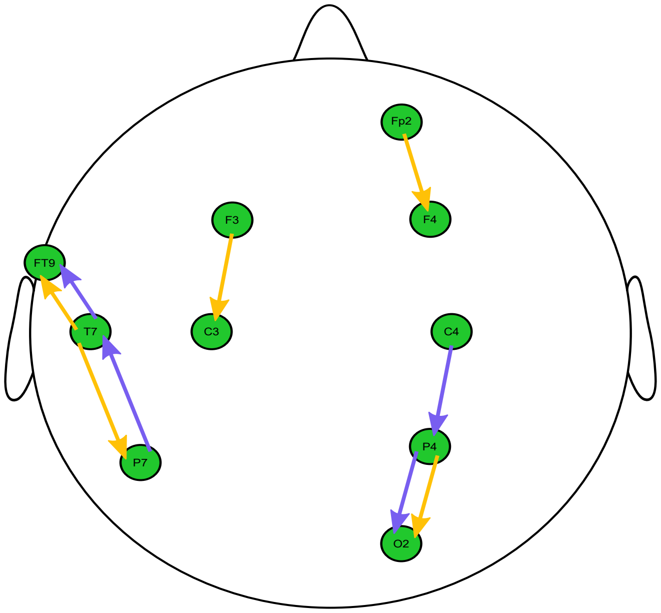

Fig. 47: **Subject 9 scalp map** Sensor pairs selected for the continuous features are shown in blue, while binary features are shown in yellow.

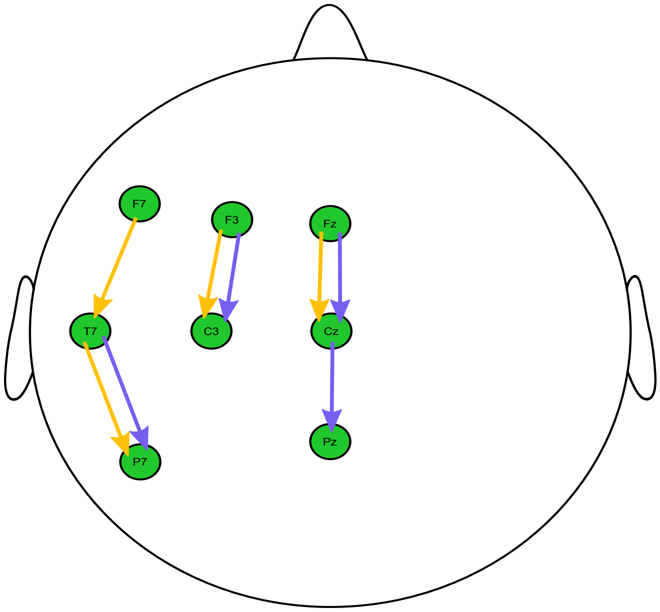

Fig. 48: **Subject 10 scalp map** Sensor pairs selected for the continuous features are shown in blue, while binary features are shown in yellow.
